# Hierarchically assembled helicates as reaction platform – from stoichiometric Diels–Alder reactions to enamine catalysis

**DOI:** 10.3762/bjoc.16.195

**Published:** 2020-09-24

**Authors:** David Van Craen, Jenny Begall, Johannes Großkurth, Leonard Himmel, Oliver Linnenberg, Elisabeth Isaak, Markus Albrecht

**Affiliations:** 1 Institute of Organic Chemistry, RWTH Aachen University, Landoltweg 1, 52074 Aachen, Germany

**Keywords:** Diels–Alder reaction, enamine catalysis, hierarchical helicates, remote-control, stereoselectivity

## Abstract

The stereoselectivity of a Diels–Alder reaction within the periphery of hierarchically assembled titanium(IV) helicates formed from mixtures of achiral, reactive and chiral, unreactive ligands was investigated in detail. Following the pathway of the chiral induction, the chiral ligands, solvents as well as substituents at the dienophile were carefully varied. Based on the results of the stoichiometric reaction, a secondary amine-catalyzed nitro-Michael reaction is performed as well which afforded reasonable diastereoselectivities.

## Introduction

**Scheme 1:**
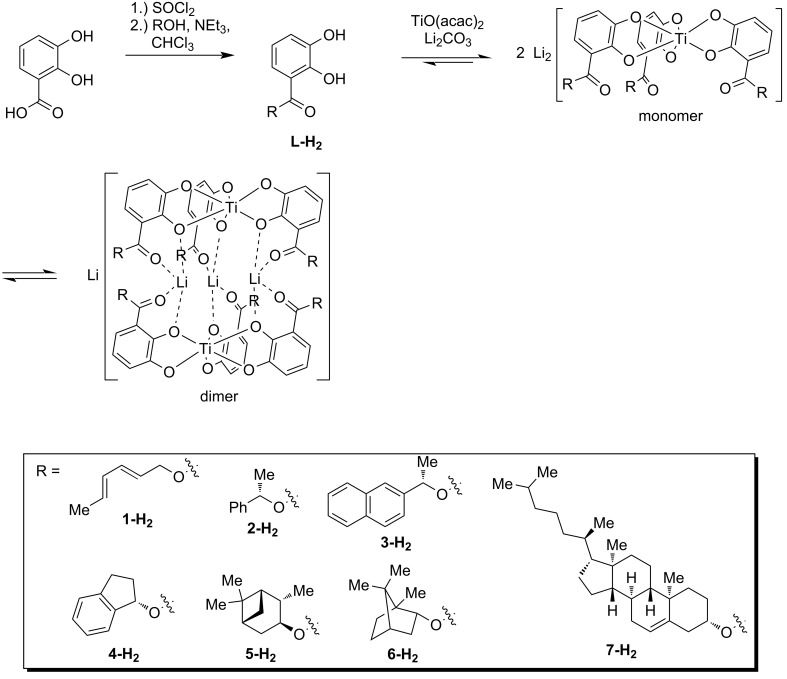
Formation of hierarchically assembled lithium-bridged titanium(IV) helicates as well as the ligands used for the stereoselective Diels–Alder reaction.

Carbon–carbon (C–C) bond-forming reactions play a key role in organic chemistry. Hereby the stereoselectivity of the reaction is highly important due to the different behavior of stereoisomers in human metabolism [1–2]. Stereocontrol was achieved either via an auxiliary [3–7] or a catalyst [8], both providing the stereoinformation necessary for induction during the C–C bond formation. Catalytic approaches for C–C bond-forming reactions even found their way into the relatively young field of supramolecular chemistry, e.g., regioselective Diels–Alder reactions within supramolecular hosts as described by Fujita et al. [9–11] or stereoselective nucleophilic substitutions by Raymond et al. [12] are important examples in this context. Recently, we described the use of hierarchically assembled helicates as templates for stereoselective Diels–Alder reactions via a post-functionalization process [13]. Catechol ligands **L-H_2_** with an ester functionality in the 3-position were prepared via conversion of the acid chloride of 2,3-dihydroxybenzoic acid to the corresponding esters. These ligands underwent a complexation with titanoyl(IV) bisacetylacetonate and lithium carbonate initially forming a mononuclear “Werner-type” triscatecholate titanium(IV) complex. Two of these monomers dimerized in a consecutive step to obtain a non-covalently linked helicate (Scheme 1). The dimerization took place via the coordination of three lithium cations acting as bridges between two monomeric complex units [13–20].

**Scheme 2:**
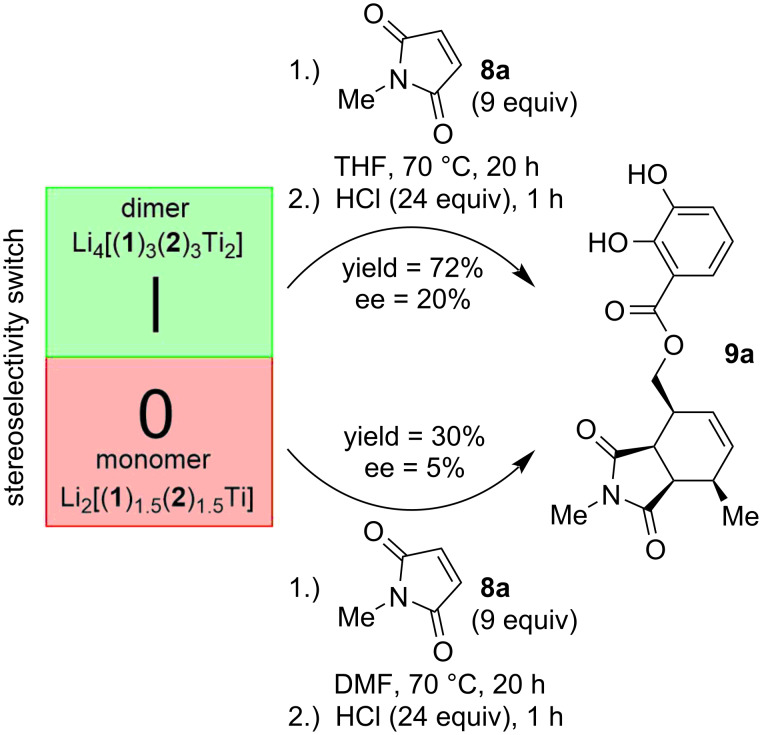
Previously reported on/off switch for “remote-controlled” [23–31] stereoselectivity of a Diels–Alder reaction by use of different solvents. The heteroleptic complexes are mixtures with an average ligand distribution as shown [13].

Enantioselectivities up to 25% ee at elevated temperature (32% ee at 0 °C) depending on the substrate were achieved in a Diels–Alder reaction by introducing two different substituted catechol ester ligands during the complex formation: (1) A diene-substituted ligand **1-H_2_** for the Diels–Alder reaction [21–22] and (2) a chiral ligand **2-H_2_** for the stereocontrol [13]. Cleaving the complex under acidic conditions resulted in the desired enantiomerically enriched product **9** and enabled the recovery of the chiral ligand **2-H_2_** (Scheme 2) [13].

The solvent choice allowed on/off-switching of the stereoselectivity of the Diels–Alder reaction. In THF the stereochemically locked dimer of the hierarchical helicate was present. Here stereoselectivity was turned on. On the other hand, the highly dynamic and fast diastereomerizing/epimerizing monomer was the major species in DMF switching off the stereoselectivity [13].

Herein we investigated the induction pathway and significantly optimized the stereoselectivity of the reaction. Furthermore, a catalytic approach was introduced which paves the way to the final goal of supramolecular stereoselective catalysis with hierarchical helicates as homogeneous catalysts.

## Results and Discussion

### Stereoselective Diels–Alder reactions in the periphery of hierarchically assembled helicates

Elucidating the induction pathway of the Diels–Alder reaction is vital for the optimization of the system described above and for the development of future processes based on the principle to use self-assembled coordination platforms (or as in the present case mixtures thereof) to control stereoselective C–C bond-forming reactions. Stereoinduction usually relies on spatial proximity of the prochiral carbon atoms and a chiral information of, e.g., a chiral auxiliary, Lewis acid or catalyst. In the previously reported system two different induction pathways were conceivable: (1) A chiral ligand is located close to the diene and controls the stereochemistry of the cycloaddition. (2) The chiral ligand controls the helicity of the helicate (ΔΔ or ΛΛ) and the helix induces the stereoselectivity of the Diels–Alder reaction.

**Scheme 3:**
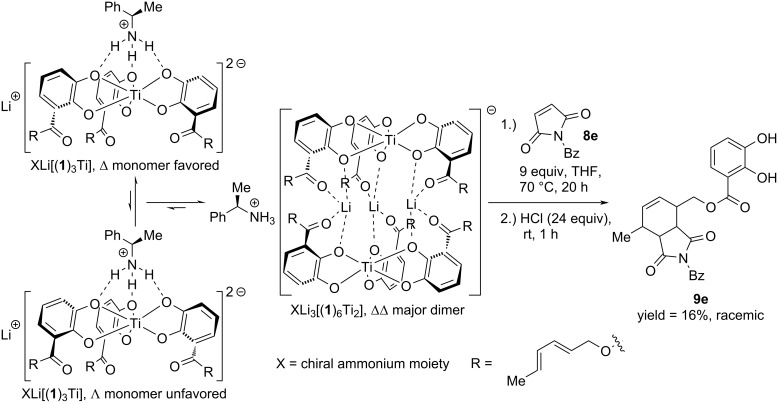
Elucidating the pathway of the stereoinduction of the Diels–Alder reaction. Ten equivalents of chiral ammonium salt are added to the hierarchical helicate in methanol and stirred for two weeks. Afterwards methanol is removed and the residue is dissolved in THF to perform a Diels–Alder reaction at the side chain.

To find out which of the induction pathways takes over the control of the Diels–Alder reaction in the periphery of the helicates, a specific helicity was induced at an achiral diene bearing helicate. It has been described before that an addition of chiral ammonium salts leads to the preference of a specific twist at the helicate [32]. As inductor, (*R*)-1-phenylethylammonium chloride was added to the racemic hexadiene-substituted helicate [Li_3_(**1**)_6_Ti_2_]^−^. The chiral salt influences the helicity of the monomeric complexes and which dimerize to the right-handed (ΔΔ) helicate [32]. As the process is slow, the mixture of the ammonium salt and complex was stirred for two weeks at room temperature in methanol. Thereafter, the solvent was removed and the Diels–Alder reaction with *N*-benzylmaleimide was performed at elevated temperature in THF. The reaction yielded the racemic product after purification. Scheme 3 is showing that the induction of stereochemistry of the Diels–Alder reaction depends on the chirality at the chiral ligand and not at the helix. This allows improvement of the stereoselectivity by using more appropriate sterically hindered or rigid chiral ligands. In addition, a solvent screening was performed in which solvents were used which favor the dimer. This is imminent for good enantioselectivities because the presence of a high amount of stereolabile monomer switches off the selectivity [13].

### Solvent dependence

Initially the solvent dependence of the stereochemical induction of the Diels–Alder reaction by the phenylethyl-derived ligand **2** was studied using *N*-benzylmaleimide (**8e**) as dienophile (Table 1). The solvents dioxane (17% ee) and acetone (14% ee) showed a slight decrease of the enantioselectivity compared to THF (21% ee). The yields of the reactions were rather moderate. On the other hand, the use of acetonitrile had no significant influence on the yield compared to acetone while the enantioselectivity dramatically dropped to 8% ee. In this case the lower selectivity correlated with the increasing lithium solvating capability of the solvent resulting in a higher proportion of the monomer and thus in lower stereoselectivities. In contrast to this, less polar solvents such as dichloromethane and chloroform resulted in increasing stereoselectivities in the Diels–Alder reaction due to their poor ability to stabilize lithium cations. Chloroform showed the best induction with 32% ee followed by dichloromethane with 25% ee, both with 50% yield (Table 1).

**Table 1:** Optimization of the stereoselectivity achieved of the Diels-Alder reaction at hierarchical helicates with solvent and chiral ligand screening.

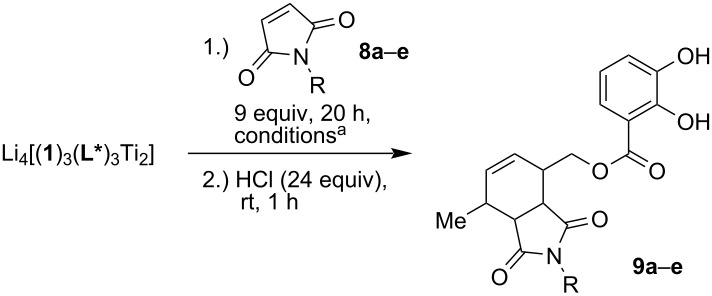							

Entry	L*	**9**	R	solvent	*T* [°C]	yield [%]	ee [%]

1	**2**	**e**	Bz	THF	70	77 [13]	21 [13]
2	**2**	**e**	Bz	dioxane	105	53	17
3	**2**	**e**	Bz	acetone	60	50	14
4	**2**	**e**	Bz	MeCN	86	44	8
5	**2**	**e**	Bz	DCM	44	50	25
6	**2**	**e**	Bz	CHCl_3_	65	50	32
7	**3**	**e**	Bz	CHCl_3_	65	71	44
8	**4**	**e**	Bz	CHCl_3_	65	64	58
9	**5**	**e**	Bz	CHCl_3_	65	61	46
10	**6**	**e**	Bz	CHCl_3_	65	64	16
11	**7**	**e**	Bz	CHCl_3_	65	11	−8
12	**4**	**a**	Me	CHCl_3_	65	76	43
13	**4**	**b**	Et	CHCl_3_	65	79	39
14	**4**	**c**	*t*-Bu	CHCl_3_	65	82	18
15	**4**	**d**	Cy	CHCl_3_	65	80	49

^a^Reactions performed in closed tubes.

### Ligand screening

In a second optimization step, the chiral ligands have been varied. An increase of stereoselectivity was achieved by using the helicates with a statistical ligand distribution Li[Li_3_(**1**)_3_(L*)_3_Ti_2_] (L* = **3**–**7**-H_2_). The given formula only describes the ratio of the ligands but in fact a statistical mixture of complexes Li[Li_3_(L*)_6_Ti_2_], Li[Li_3_(**1**)(L*)_5_Ti_2_], Li[Li_3_(**1**)_2_(L*)_4_Ti_2_], Li[Li_3_(**1**)_3_(L*)_3_Ti_2_], Li[Li_3_(**1**)_4_(L*)_2_Ti_2_], Li[Li_3_(**1**)_5_(L*)_1_Ti_2_], and Li[Li_3_(**1**)_6_Ti_2_] is present. Expanding the aromatic unit to a naphthyl group in **3-H_2_** resulted in an increase of the enantioselectivity to 44% ee. Even better selectivities were obtained with **4-H_2_** bearing an indanyl [33–34] substituent which combines a stereogenic center implemented in a ring system providing rigidity as well as an aromatic residue. The enantioselectivity increased to 58% ee (Table 1).

Besides the aromatic ligands, terpenyl-substituted ligands were investigated, too. The largest ligand **7-H_2_** with a cholesteryl moiety favored the opposite enantiomer, however, only with −8% ee in only 11% yield. The low yield may be attributed to the poor solubility of the helicate. The other terpene [35–36] derived systems Li[Li_3_(**1**)_3_(**5**)_3_Ti_2_] and Li[Li_3_(**1**)_3_(**6**)_3_Ti_2_] showed a different behavior. The (1*S*,2*S*,3*S*,5*R*)-3-pinanyl-substituted Li[Li_3_(**1**)_3_(**5**)_3_Ti_2_] yielded 46% ee, while the complex bearing a ʟ*-*(−)-borneyl residue Li[Li_3_(**1**)_3_(**6**)_3_Ti_2_] showed only 16% ee. The yields were higher than 60%. A possible reason for the significant drop in enantioselectivity by switching from ligand **5** to **6** was due to the different dimerization behavior. The homoleptic helicate Li[Li_3_(**6**)_6_Ti_2_] shows a lower dimerization tendency compared to Li[Li_3_(**5**)_6_Ti_2_] [35–36]. Thus, the higher amount of undesired monomer in solution of Li[Li_3_(**1**)_3_(**6**)_3_Ti_2_] resulted in a partial switch-off of the stereoselectivity.

### Screening of the dienophile

The variation of the dienophile was studied in chloroform using the helicate Li_4_[(**1**)_3_(**4**)_3_Ti_2_]. *N*-Maleimides **8a** and **8b** with a methyl and an ethyl residue showed higher yields and a lower induction in comparison to the benzyl derivative **8e** with 43% ee and 39% ee (Table 1). The poorest result was obtained by using dienophile **8c** with a *tert*-butyl substituent (82% yield, 18% ee). This maleimide gave the lowest induction in our previous work, too [13]. Thus, no improvement was made in comparison to the 15% ee [13] achieved with chiral ligand **2**-**H_2_** in THF as solvent. The cyclohexyl-substituted dienophile **8d** showed a higher induction (49% ee and 80% yield) than **8a** and **8b**, but could not reach the results of **8e**. The described optimization of the reaction conditions based on solvent, chiral ligand, and substituent at the dienophile resulted in a nearly threefold increase of the enantioselectivity compared to the earlier described results [13].

The screening showed the opportunity to use hierarchically formed helicates with mixtures of ligands as platforms to control the stereochemistry of C–C bond-forming reactions. However, it would be of great advantage to transfer the findings to catalytic C–C bond-forming reactions which are catalyzed by hierarchical helicates containing chiral ligands for stereocontrol and achiral catalytically active ligands.

### Enamine-catalyzed nitro-Michael reactions

**Scheme 4:**
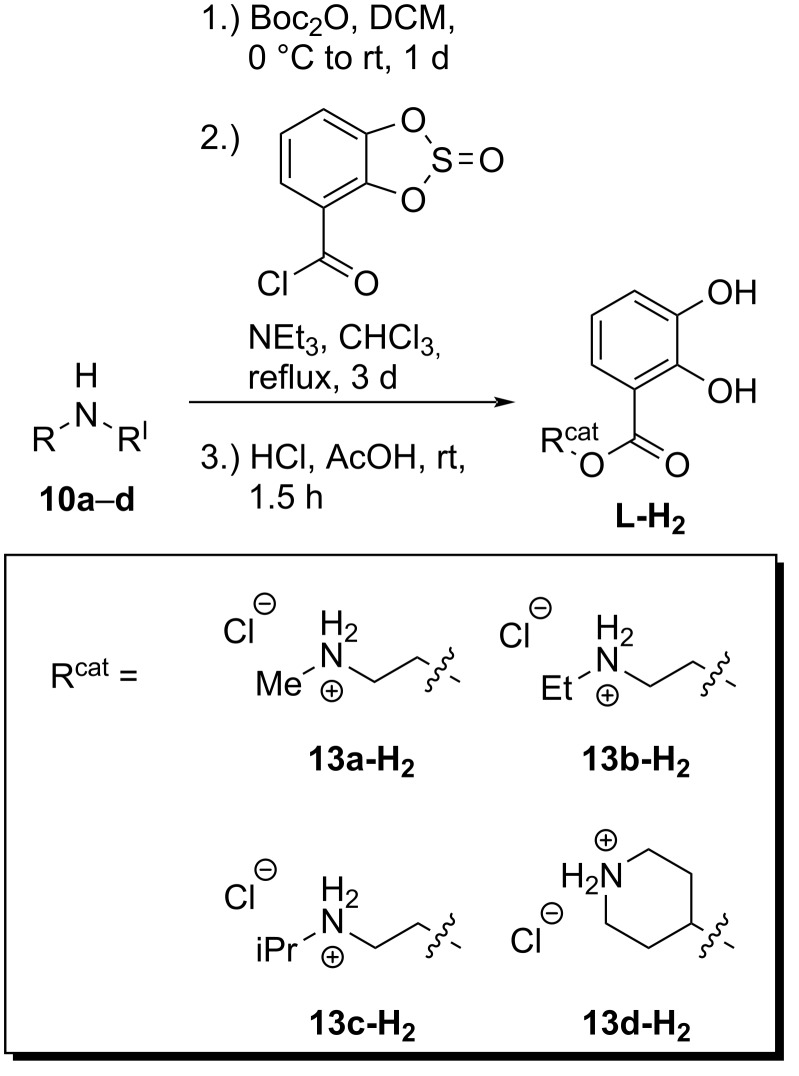
Synthesis of the ligands with secondary amine-containing substituents.

The nitro-Michael reaction [37–40] seemed to be suitable to be performed at hierarchically assembled helicates due to the reaction’s “benchmark character” [41]. Therefore, ligands bearing secondary amine residues were introduced instead of the diene ligands. Again helicates with a statistical distribution of chiral ligands and of the new amine ligands in the complex were investigated as catalysts. The ligands with potential catalytic activity were synthesized in a three-step approach (Scheme 4). Initially the amino alcohols **10a**–**d** were protected with a Boc group [42–43]. Esterification of the protected alcohols **11a–d** [33,44] with 2,3-dioxosulfinylbenzoyl chloride obtained from 2,3-dihydroxybenzoic acid and thionyl chloride afforded the *N*-Boc-substituted catechol ligands **12a**–**d** [33,36]. They were deprotected under acidic conditions with hydrochloric acid yielding ligands **13a–d-H_2_** [33,36] as ammonium chloride salts.

The obtained ligands **13a–d-H_2_** were used together with the chiral ligands **2,4,5-H_2_** for the formation of hierarchical helicates with a statistical ligand ratio which were formed from 1 equivalent of **13-H_2_** and 5 equivalents of **2-H_2_**, **4-H_2_**, and **5-H_2_**.

The catalytic activity of the amine ligands was tested first by using the uncoordinated ligand **13a-H_2_** substituted with a *N*-methylethylamine moiety. The reaction was performed in DMSO-*d*_6_ due to solubility limitations of the ligand. Fast and easy measurement of the yield and the diastereoselectivity was possible by NMR spectroscopy. The nitro-Michael reaction of 3 equivalents propanal (**14**) and β-nitrostyrene (**15**) with 25 mol % of **13a-H_2_** after 2 days at room temperature resulted in 45% yield of product **16** and a nearly 1:1 diastereomeric ratio (Table 2).

**Table 2:** Enamine-catalyzed nitro-Michael reaction with hierarchically assembled helicates.^a^

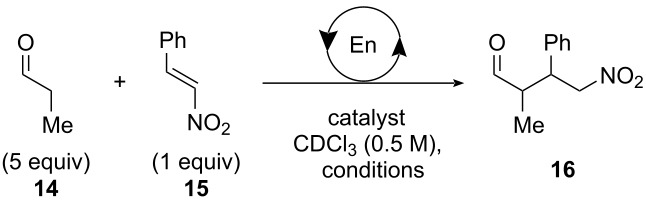						

Entry	catalyst	mol %	*T* [°C]	*t* [d]	yield [%]	dr

1^b^	**13a-H_2_**	25	rt	2	45^c^	52:48^c^
2	Li_4_[(**13a**)_1_(**2**)_5_Ti_2_]	15	rt	3	0	–
3	Li_4_[(**13b**)_1_(**2**)_5_Ti_2_]	15	rt	3	88	83:17
4	Li_4_[(**13b**)_1_(**2**)_5_Ti_2_]	15	0	7	71	87:13
5	Li_4_[(**13b**)_1_(**2**)_5_Ti_2_]	7.5	70	1	48	65:35
6	Li_4_[(**13c**)_1_(**2**)_5_Ti_2_]	15	rt	3	0	–
7	Li_4_[(**13d**)_1_(**2**)_5_Ti_2_]	15	rt	3	0	–
8	Li_4_[(**13b**)_1_(**4**)_5_Ti_2_]	15	rt	3	13	80:20
9	Li_4_[(**13b**)_1_(**5**)_5_Ti_2_]	15	rt	3	27	80:20

^a^No enantioselectivity was achieved. ^b^Reaction was performed in DMSO-*d*_6_ (0.26 M) due to solubility limitations of the free ligand with 3 equiv of propanal. ^c^Values determined by integration of the crude NMR spectrum of the reaction.

Catalysis at the “statistical” helicates was carried out with 5 equivalents of propanal (**14**) in order to gain a higher conversion. Beside a significant control over the diastereomeric ratio no enantioselectivity was achieved with helicates as catalysts. Catalysts at concentrations of 15 mol % were used in CDCl_3_ at room temperature and 0 °C with three or seven days of reaction time. The conversion was controlled by NMR spectroscopy and TLC. The helicate Li_4_[(**13a**)_1_(**2**)_5_Ti_2_] did not lead to any conversion at room temperature (Table 2). The catalyst Li_4_[(**13b**)_1_(**2**)_5_Ti_2_] with an ethyl-substituted amine worked well resulting in 88% yield and 66% de at room temperature. The diastereomeric excess increased slightly to a maximum of 74% de (dr 87:13) at 0 °C. A dramatic decrease to 30% de was observed by lowering the catalyst loading to 7.5 mol % while increasing the temperature to 70 °C. No enantioselectivity was observed using the helicate Li_4_[(**13b**)_1_(**2**)_5_Ti_2_] as catalyst. The helicates Li_4_[(**13c**)_1_(**2**)_5_Ti_2_] and Li_4_[(**13d**)_1_(**2**)_5_Ti_2_] with an isopropyl-substituted ethylamine and a cyclic secondary amine ligand as catalytically active unit showed no conversion in the nitro-Michael reaction. Solubility problems were the supposed reason for this observation. Thus, the amine ligand **13b-H_2_** seemed to be an appropriate component to make helicates from ligand mixtures which possess catalytic activity.

Exchange of the chiral ligand **2** by other chiral ones resulted in the corresponding complexes Li_4_[(**13b**)_1_(**4**)_5_Ti_2_] and Li_4_[(**13b**)_1_(**5**)_5_Ti_2_], but did not lead to a control of enantioselectivity. A reasonable diastereoselectivity of 60% de was observed for both catalysts. The limited solubility of these complexes caused a significant reduction of the yield at room temperature and due to this the reaction was not performed at lower temperatures.

## Conclusion

An optimization of the Diels–Alder reaction taking place in the periphery of hierarchically assembled helicates was carried out. It was based on the elucidated induction pathway showing that the stereoselectivity was due to the proximity of the chiral units of ligand **2** to the diene unit. The helicity of the helicate did not have a significant influence. After optimization of solvent, chiral ligand, and substituent at the dienophile stereoselectivity was nearly tripled. Up to 58% ee was achieved in the Diels–Alder reaction in chloroform with the indanyl-substituted chiral ligand **4-H_2_** and *N-*benzylmaleimide (**8e**) as the dienophile.

In addition, the transition from the stoichiometric Diels–Alder reaction to a catalytic nitro-Michael reaction was described utilizing secondary amine ligands as catalysts. Only amine ligand **13b-H_2_** seemed suitable in the catalysis with the corresponding statistical helicates. With other complexes solubility problems arose. Li_4_[(**13b**)_1_(**2**)_5_Ti_2_] was the most efficient catalyst discussed in this study and provided good yields of up to 88% at room temperature. Suitable diastereoselectivities were obtained with up to 74% de (dr 87:13) at 0 °C and 66% de (dr 83:17) at room temperature. Enantioselectivity was not achieved even with the chiral ligands **4-H_2_** and **5-H_2_**.

Nevertheless, the successful implementation of diastereoselective catalysis by hierarchically assembled helicates was a big step forward and will draw our focus on the development of new systems possessing catalytic activity with improved solubility.

## Supporting Information

File 1 Synthetic procedures, characterization data, SFC and HPLC conditions and copies of ^1^H and ^13^C NMR spectra of new compounds.
