# Supplementary material for: Hierarchically assembled helicates as reaction platform – from stoichiometric Diels–Alder reactions to enamine catalysis
Source: Beilstein J Org Chem. 2020 Sep 24;16:2338–45. doi: 10.3762/bjoc.16.195 (PMC7522461; doi:10.3762/bjoc.16.195)
Supplement: File 1 — Synthetic procedures, characterization data, SFC and HPLC conditions and copies of 1H and 13C NMR spectra of new compounds. [file Beilstein_J_Org_Chem-16-2338-s001.pdf]

BEILSTEIN JOURNAL OF ORGANIC CHEMISTRY

# Supporting Information

for

# Hierarchically assembled helicates as reaction platform – from stoichiometric Diels–Alder reactions to enamine catalysis

David Van Craen, Jenny Begall, Johannes Großkurth, Leonard Himmel, Oliver Linnenberg, Elisabeth Isaak and Markus Albrecht

*Beilstein J. Org. Chem.* **2020,** *16,* 2338–2345. doi:10.3762/bjoc.16.195

## Synthetic procedures, characterization data, SFC and HPLC conditions and copies of $^{1}H$ and $^{13}C$ NMR spectra of new compounds

License and Terms: This is a supporting information file under the terms of the Creative Commons Attribution License (https://creativecommons.org/licenses/by/4.0). Please note that the reuse, redistribution and reproduction in particular requires that the authors and source are credited.
The license is subject to the *Beilstein Journal of Organic Chemistry* terms and conditions: (https://www.beilstein-journals.org/bjoc)

**Materials and methods:** Chemicals were obtained from Sigma Aldrich, Alfa Aesar, and Acros. Solvents were purified via distillation before use. TLC plates were obtained from Merck (Silica 60, F 254, 0.25 mm thickness) and the visualization was achieved with UV light (254 nm) and $KMnO_4$ solution. NMR spectra were measured at Mercury 300, Varian VNMRS 400, and 600 devices. ESI mass spectrometry was carried out at a LTQ Orbitrap XL. IR spectra were obtained from a Perkin-Elmer Spektrum 100 spectrometer and melting points were determined at a Büchi B-54 device. Elemental analysis was carried out at a Heraeus CHN-O-Rapid device.

**Synthetic procedure for ligands:** 2,3-Dihydroxybenzoic acid is converted into the corresponding acid chloride via refluxing with thionyl chloride (30 equiv). The excess of thionyl chloride is removed under reduced pressure after full conversion (change from a dispersion to a solution) was observed. The following esterification takes place in chloroform (0.2 mol $L^{-1}$). The initially obtained 2,3-dioxosulfinylbenzoyl chloride is not further purified and dissolved in four fifths of the necessary chloroform. A solution of the corresponding alcohol (3 equiv) and triethylamine (6 equiv) dissolved in one fifths of the chloroform is added and the mixture is refluxed for three days. The ligand **L-H$_2$** is obtained after washing the reaction mixture with saturated $NaHCO_3$ solution, drying over $MgSO_4$ and purification via column chromatography with silica gel 60 (35–70 µm).

**Synthetic procedure for statistical complexes:** The corresponding achiral ligand (3 equiv) and chiral ligand (3 equiv) are dissolved in methanol (0.02 mol $L^{-1}$). The complexation is achieved by adding $TiO(acac)_2$ (2 equiv) and $Li_2CO_3$ (2 equiv) followed by stirring the reaction mixture over night. No purification is necessary to obtain the statistical complexes except for removing the solvent under reduced pressure. The complexes consisting of the amine ligands are obtained by mixing the amine ligand (1 equiv) with the chiral ligand (5 equiv) in methanol/chloroform 50:50 (0.02 mol $L^{-1}$) as solvent mixture. The rest of the procedure follows the one described above.

**Diels–Alder reactions at hierarchical helicates as platform:** Complex $Li_4[(\mathbf{1})_3(\mathbf{L^*})_3Ti_2]$ (200 mg, 1 equiv) is post-functionalized with dienophile **8** (9 equiv) in the corresponding solvent (2 mL) under the given conditions in a closed reaction tube. Hydrochloric acid (37 %, 24 equiv) mixed with 0.5 mL water is added to the solution, if the solvent is THF, dioxane, acetone, or acetonitrile. If dichloromethane or chloroform is used, the solvent is removed and the residue is dissolved in THF before hydrochloric acid (37 %, 24 equiv) mixed with 0.5 mL water is added. The cleavage is finished after mixing the solution for one hour. The mixture is diluted with dichloromethane (40 mL), washed with saturated sodium bicarbonate solution (4 mL), and dried over $MgSO_4$. The product is obtained via column chromatography according to the literature[1] after removal of the solvent.

**Synthesis of *N*-Boc-protected alcohols:** Amino alcohol **10** (1.2 equiv) is dissolved in dichloromethane (0.67 mol $L^{-1}$) and cooled to 0 °C. A solution of Boc anhydride (1 equiv) in dichloromethane (0.33 mol $L^{-1}$) is added dropwise in one hour to the amino alcohol solution. The mixture is stirred at room temperature overnight and the solvent is removed under reduced pressure. Purification is achieved via column chromatography with silica gel 60 (35–70 µm).

**Cleavage of the Boc group resulting in ligands 13-H$_2$:** Boc-protected catechol ester **12** (1 equiv) is poured into glacial acetic acid and hydrochloric acid (37%, 10 equiv) is added to this solution. The reaction mixture is stirred for one hour. If the product

precipitates, it is filtered off and washed with diethyl ether. If precipitation does not occur, the solvent is removed under reduced pressure and the product is dried.

**Nitro-Michael reaction with hierarchical helicates as catalysts:** The catalyst $Li_4[(\mathbf{13})_1(\mathbf{L^*})_5Ti_2]$ and β-nitrostyrene (52.2 mg, 0.35 mmol, 1 equiv) are dissolved in deuterated chloroform (0.75 mL). Propanal (125 µL, 1.75 mmol, 5 equiv) is added and the reaction mixture is stirred under the given conditions. The conversion is checked via TLC and $^1H$ NMR spectroscopy afterwards. The product is isolated via column chromatography (pentane/diethylether 3:1, $R_f$ = 0.14).

The compounds **1-H2**[1], **2-H2**[2], **3-H2**[3], (*S*)-1-indanole[4], **5-H2**[5], **6-H2**[5], $Li_4[(\mathbf{1})_3(\mathbf{2})_3Ti_2]$[1], $Li_4[(\mathbf{1})_6Ti_2]$[1], **9a-e**[1], and **16**[7,8,9] are already described in the literature. Additionally, compounds **4-H2**[10], **11a**[6,10], **11b**[6,10], **11c**[10], **11d**[10,11], **12a**[12], **12b–d**[10], **13a-H2**[12], **13b–d-H2**[10] and their corresponding complexes[10] are known.

**(*S*)-1-Indanyl 2,3-dihydroxybenzoate (4-H2):** Two equiv alcohol and four equiv triethylamine are used in this case. Y = 50 % (409 mg, 1.51 mmol, colorless oil). $\mathbf{R_f}$ = 0.21 (pentane/ethylacetate 18:1). $^1$**H NMR** (400 MHz, $CDCl_3$, 25 °C): δ = 11.02 (s, 1H, O*H*), 7.48 (d, 1H, *J* = 7.5 Hz, $H_{arom.}$), 7.35-7.28 (m, 3H, $H_{arom.}$), 7.27-7.21 (m, 1H, $H_{arom.}$), 7.09-7.05 (m, 1H, $H_{arom.}$), 6.73 (t, 1H, *J* = 8.0 Hz, $H_{arom.}$), 6.45 (dd, 1H, *J* = 7.0, 3.7 Hz, $CO_2CH$), 5.62 (s, 1H, O*H*), 3.24-3.14 (m, 1H, $H_{cycl.}$), 2.95 (ddd, 1H, *J* = 16.1, 8.5, 4.8 Hz, $H_{cycl.}$), 2.66-2.56 (m, 1H, $H_{cycl.}$), 2.31-2.22 (m, 1H, $H_{cycl.}$) ppm. $^{13}$**C NMR** (101 MHz, $CDCl_3$, 25 °C): δ = 170.3 ($CO_2CH$), 148.9 ($C_{arom.}$), 145.0 ($C_{arom.}$), 144.5 ($C_{arom.}$), 140.2 ($C_{arom.}$), 129.3 ($C_{arom.}$), 126.8 ($C_{arom.}$), 125.7 ($C_{arom.}$), 124.9 ($C_{arom.}$), 120.7 ($C_{arom.}$), 119.7 ($C_{arom.}$), 119.0 ($C_{arom.}$), 112.7 ($C_{arom.}$), 79.8 ($CO_2CH$), 32.3 ($C_{cycl.}$), 30.2 ($C_{cycl.}$) ppm. **MS** (positive ESI-FTMS, MeOH, acidified): m/z (%) = 293.07837 (95, [M+Na$^+$], $C_{16}H_{14}O_4Na^+$, calc. 293.07843). **IR** (in KBr): ṽ (cm$^{-1}$) = 3793, 2971, 2105, 1739, 1367, 1222, 1009, 739. **Elemental analysis** $C_{16}H_{14}O_4$: calc. C 71.10 %, H 5.22 %; found C 71.38 %, H = 5.24 %.

**Cholesteryl 2,3-dihydroxybenzoate (7-H2):** Three equiv alcohol and triethylamine were used in this case. Y = 53 % (1.38 g, 2.64 mmol, colorless solid). $\mathbf{R_f}$ = 0.54 (dichloromethane). $^1$**H NMR** (600 MHz, $CDCl_3$, 25 °C): δ = 11.07 (s, 1H, O*H*), 7.38 (dd, 1H, *J* = 8.0, 1.4 Hz, $H_{arom.}$), 7.13-7.02 (m, 1H, $H_{arom.}$), 6.79 (t, 1H, *J* = 8.0 Hz, $H_{arom.}$), 5.62 (s, 1H, O*H*), 5.43 (d, 1H, *J* = 5.1 Hz, $CH_{double}$), 4.93-4.83 (m, 1H, $CO_2CH$), 2.52-2.43 (m, 2H), 2.06-1.91 (m, 4H), 1.88-1.72 (m, 2H), 1.62-1.45 (m, 6H), 1.42-1.30 (m, 3H), 1.29-1.08 (m, 8H), 1.07 (s, 3H, $CCH_3$), 1.05-0.96 (m, 3H), 0.92 (d, 3H, *J* = 6.6 Hz, $CHCH_3$), 0.87 (dd, 6H, *J* = 6.7, 2.8 Hz, $CH(CH_3)_2$), 0.69 (s, 3H, $CCH_3$) ppm. $^{13}$**C NMR** (151 MHz, $CDCl_3$, 25 °C): δ = 170.0 ($CO_2CH$), 149.1 ($C_{arom.}$), 145.1 ($C_{arom.}$), 139.4 ($C_{double}$), 123.3 ($CH_{double}$), 120.7 ($C_{arom.}$), 119.7 ($C_{arom.}$), 119.2 ($C_{arom.}$), 113.0 ($C_{arom.}$), 75.7 ($CO_2CH$), 56.8 (*C*H), 56.3 (*C*H), 50.2 (*C*H), 42.5 (*C*), 39.9 ($CH_2$), 39.7 ($CH_2$), 38.2 ($CH_2$), 37.1 ($CH_2$), 36.8 (*C*), 36.3 ($CH_2$), 35.9 (*C*H), 32.1 ($CH_2$), 32.0 (*C*H), 28.4 ($CH_2$), 28.2 (*C*H), 27.9 ($CH_2$), 24.4 ($CH_2$), 24.0 ($CH_2$), 23.0 (1×$CH_3$ of $CH(CH_3)_2$), 22.7 (1×$CH_3$ of $CH(CH_3)_2$), 21.2 ($CH_2$), 19.5 ($CCH_3$), 18.9 ($CHCH_3$), 12.0 ($CCH_3$) ppm. **MS** (EI, 70 eV): m/z (%) = 522.6 ([M$^+$], $C_{34}H_{50}O_4^+$). **IR** (in KBr): ṽ (cm$^{-1}$) = 3540, 3049, 2945, 2865, 2320, 2164, 2081, 1995, 1923, 1743, 1666, 1613, 1522, 1465, 1408, 1374, 1303, 1260, 1234, 1199, 1152, 1064, 1027, 984, 922, 886, 843, 801, 783, 755, 704. **Elemental analysis** $C_{34}H_{50}O_4$: calc. C 78.12 %, H 9.64 %; found C 77.84 %, H 9.22 %.

**$\mathbf{Li_4[(1)_3(3)_3Ti_2]}$:** Y = quantitative (494.8 mg, red solid). **MS** (negative ESI-FTMS, MeOH): m/z (%) = 1509.43884 (15, $[Li_3[(\mathbf{1})_6Ti_2]]^-$, $C_{78}H_{72}O_{24}Li_3Ti_2^-$, calc. 1509.38581),

1583.45935 (60, [$Li_3$[(**1**)$_5$(**3**)$_1$$Ti_2$]]$^-$, $C_{84}H_{74}O_{24}Li_3Ti_2^-$, calc. 1583.40146), 1657.47815 (99, [$Li_3$[(**1**)$_4$(**3**)$_2$$Ti_2$]]$^-$, $C_{90}H_{76}O_{24}Li_3Ti_2^-$, calc. 1657.41711), 1731.49585 (100, [$Li_3$[(**1**)$_3$(**3**)$_3$$Ti_2$]]$^-$, $C_{96}H_{78}O_{24}Li_3Ti_2^-$, calc. 1731.43276), 1805.51208 (66, [$Li_3$[(**1**)$_2$(**3**)$_4$$Ti_2$]]$^-$, $C_{102}H_{80}O_{24}Li_3Ti_2^-$, calc. 1805.44841), 1879.52905 (20, [$Li_3$[(**1**)$_1$(**3**)$_5$$Ti_2$]]$^-$, $C_{108}H_{82}O_{24}Li_3Ti_2^-$, calc. 1879.46406).

**$Li_4$[(1)$_3$(4)$_3$$Ti_2$]:** Y = quantitative (577.7 mg, red solid). **MS** (negative ESI-FTMS, MeOH): m/z (%) = 1509.44092 (21, [$Li_3$[(**1**)$_6$$Ti_2$]]$^-$, $C_{78}H_{72}O_{24}Li_3Ti_2^-$, calc. 1509.38581), 1545.44312 (69, [$Li_3$[(**1**)$_5$(**4**)$_1$$Ti_2$]]$^-$, $C_{81}H_{72}O_{24}Li_3Ti_2^-$, calc. 1545.38581), 1581.44385 (100, [$Li_3$[(**1**)$_4$(**4**)$_2$$Ti_2$]]$^-$, $C_{84}H_{72}O_{24}Li_3Ti_2^-$, calc. 1581.38581), 1617.44373 (95, [$Li_3$[(**1**)$_3$(**4**)$_3$$Ti_2$]]$^-$, $C_{87}H_{72}O_{24}Li_3Ti_2^-$, calc. 1617.38581), 1653.44324 (64, [$Li_3$[(**1**)$_2$(**4**)$_4$$Ti_2$]]$^-$, $C_{90}H_{72}O_{24}Li_3Ti_2^-$, calc. 1653.38581), 1689.44287 (22, [$Li_3$[(**1**)$_1$(**4**)$_5$$Ti_2$]]$^-$, $C_{93}H_{72}O_{24}Li_3Ti_2^-$, calc. 1689.38581).

**$Li_4$[(1)$_3$(5)$_3$$Ti_2$]:** Y = quantitative (359.8 mg, red solid). **MS** (negative ESI-FTMS, MeOH): m/z (%) = 1509.42029 (20, [$Li_3$[(**1**)$_6$$Ti_2$]]$^-$, $C_{78}H_{72}O_{24}Li_3Ti_2^-$, calc. 1509.38581), 1565.48572 (62, [$Li_3$[(**1**)$_5$(**5**)$_1$$Ti_2$]]$^-$, $C_{82}H_{80}O_{24}Li_3Ti_2^-$, calc. 1565.44841), 1621.54980 (93, [$Li_3$[(**1**)$_4$(**5**)$_2$$Ti_2$]]$^-$, $C_{86}H_{88}O_{24}Li_3Ti_2^-$, calc. 1621.51101), 1677.61292 (100, [$Li_3$[(**1**)$_3$(**5**)$_3$$Ti_2$]]$^-$, $C_{90}H_{96}O_{24}Li_3Ti_2^-$, calc. 1677.57361), 1733.67554 (82, [$Li_3$[(**1**)$_2$(**5**)$_4$$Ti_2$]]$^-$, $C_{94}H_{104}O_{24}Li_3Ti_2^-$, calc. 1733.63621), 1789.73779 (45, [$Li_3$[(**1**)$_1$(**5**)$_5$$Ti_2$]]$^-$, $C_{98}H_{112}O_{24}Li_3Ti_2^-$, calc. 1789.69881), 1845.80078 (10, [$Li_3$[(**5**)$_6$$Ti_2$]]$^-$, $C_{102}H_{120}O_{24}Li_3Ti_2^-$, calc. 1845.76141).

**$Li_4$[(1)$_3$(6)$_3$$Ti_2$]:** Y = quantitative (599.1 mg, red solid). **MS** (negative ESI-FTMS, MeOH): m/z (%) = 1509.42932 (22, [$Li_3$[(**1**)$_6$$Ti_2$]]$^-$, $C_{78}H_{72}O_{24}Li_3Ti_2^-$, calc. 1509.38581), 1565.49512 (71, [$Li_3$[(**1**)$_5$(**6**)$_1$$Ti_2$]]$^-$, $C_{82}H_{80}O_{24}Li_3Ti_2^-$, calc. 1565.44841), 1621.55920 (100, [$Li_3$[(**1**)$_4$(**6**)$_2$$Ti_2$]]$^-$, $C_{86}H_{88}O_{24}Li_3Ti_2^-$, calc. 1621.51101), 1677.62231 (99, [$Li_3$[(**1**)$_3$(**6**)$_3$$Ti_2$]]$^-$, $C_{90}H_{96}O_{24}Li_3Ti_2^-$, calc. 1677.57361), 1733.68555 (85, [$Li_3$[(**1**)$_2$(**6**)$_4$$Ti_2$]]$^-$, $C_{94}H_{104}O_{24}Li_3Ti_2^-$, calc. 1733.63621), 1789.74841 (58, [$Li_3$[(**1**)$_1$(**6**)$_5$$Ti_2$]]$^-$, $C_{98}H_{112}O_{24}Li_3Ti_2^-$, calc. 1789.69881), 1845.81116 (23, [$Li_3$[(**6**)$_6$$Ti_2$]]$^-$, $C_{102}H_{120}O_{24}Li_3Ti_2^-$, calc. 1845.76141).

**$Li_4$[(1)$_3$(7)$_3$$Ti_2$]:** The complexation was performed in methanol/chloroform (50:50). Y = quantitative (312.1 mg, red solid). **MS** (negative ESI-FTMS, MeOH): m/z (%) = 1509.43311 (100, [$Li_3$[(**1**)$_6$$Ti_2$]]$^-$, $C_{78}H_{72}O_{24}Li_3Ti_2^-$, calc. 1509.38581), 1797.72168 (34, [$Li_3$[(**1**)$_5$(**7**)$_1$$Ti_2$]]$^-$, $C_{99}H_{108}O_{24}Li_3Ti_2^-$, calc. 1797.66751), 2086.01392 (10, [$Li_3$[(**1**)$_4$(**7**)$_2$$Ti_2$]]$^-$, $C_{120}H_{144}O_{24}Li_3Ti_2^-$, calc. 2085.94921).

***tert*-Butyl (2-hydroxyethyl)(isopropyl)carbamate (11c):** Y = 88 % (5.36 g, 26.4 mmol, colorless oil). **$R_f$** = 0.44 (dichloromethane/MeOH 30:1). **$^1$H NMR** (600 MHz, $CDCl_3$, 25 °C): δ = 4.11 (br, 1H, C*H*($CH_3$)$_2$), 3.66 (t, 2H, *J* = 5.6 Hz, C*H$_2$*), 3.25 (br, 2H, C*H$_2$*), 1.43 (s, 9H, C(C*H$_3$*)$_3$), 1.09 (d, 6H, *J* = 6.9 Hz, CH(C*H$_3$*)$_2$) ppm. The hydroxyl signal is not observed. **$^{13}$C NMR** (151 MHz, $CDCl_3$, 25 °C): δ = 157.7 (*C*O$_2$C($CH_3$)$_3$), 80.1 (*C*H($CH_3$)$_2$ or *C*($CH_3$)$_3$), 64.0 (*C*H$_2$), 47.9 (*C*H($CH_3$)$_2$ or *C*($CH_3$)$_3$), 44.8 (*C*H$_2$), 28.4 (C(*C*H$_3$)$_3$), 20.7 (CH(*C*H$_3$)$_2$) ppm. **MS** (positive ESI-FTMS, MeOH): m/z (%) = 226.14088 (100, [M+Na$^+$], $C_{10}H_{21}NO_3Na^+$, calc. 226.14136). **IR** (in KBr): ṽ (cm$^{-1}$) = 3436, 2973, 2323, 2090, 1669, 1550, 1460, 1405, 1361, 1250, 1164, 1119, 1037, 957, 896, 771, 697. **Elemental analysis** $C_{10}H_{21}NO_3$: calc. C 59.09 %, H 10.41 %, N 6.89 %; found C 58.64 %, H 10.56 %, N 7.39 %.

***tert*-Butyl 4-hydroxypiperidine-1-carboxylate (11d):** Y = quantitative (6.06 g, 30.1 mmol, colorless solid). **$R_f$** = 0.16 (dichloromethane/MeOH 20:1). **Mp** = 67 °C -

69 °C (dichloromethane). **$^1$H NMR** (400 MHz, $CDCl_3$, 25 °C): δ = 3.86-3.75 (m, 3H, $H_{cycl.}$), 2.98 (ddd, 2H, *J* = 13.4, 9.8, 3.3 Hz, $H_{cycl.}$), 1.97-1.91 (m, 1H, $H_{cycl.}$), 1.85-1.76 (m, 2H, $H_{cycl.}$), 1.47-1.36 (m, 10H, C(C*H*$_3$)$_3$ and $H_{cycl.}$) ppm. The hydroxyl group is not visible. **$^{13}$C NMR** (101 MHz, $CDCl_3$, 25 °C): δ = 155.0 (*C*O$_2$C(CH$_3$)$_3$), 79.7 (*C*(CH$_3$)$_3$), 67.8 ($C_{cycl.}$), 41.4 (2×$C_{cycl.}$), 34.3 (2×$C_{cycl.}$), 28.6 (C(*C*H$_3$)$_3$) ppm. **MS** (positive ESI-FTMS, MeOH): m/z (%) = 240.15611 (100, [M+K$^+$], $C_{10}H_{19}NO_3K^+$, calc. 240.09965). **IR** (in KBr): ṽ (cm$^{-1}$) = 3464, 2930, 2317, 2069, 1929, 1664, 1430, 1249, 1158, 1071, 852, 765. **Elemental analysis** $C_{10}H_{19}NO_3$: calc. C 59.68 %, H 9.52 %, N 6.96 %; found C 59.63 %, H 9.56 %, N 6.84 %.

**2-((*tert*-Butoxycarbonyl)(methyl)amino)ethyl 2,3-dihydroxybenzoate (12a):** Y = 79 % (1.23 g, 3.95 mmol, colorless solid). **$R_f$** = 0.32 (pentane /ethylacetate 4:1). **Mp** = 104 °C - 105 °C (last solvent: dichloromethane). **$^1$H NMR** (400 MHz, $CDCl_3$, 50 °C): δ = 10.74 (s, 1H, O*H*), 7.34 (dd, 1H, *J* = 7.9, 1.3 Hz, $H_{arom.}$), 7.08 (d, 1H, *J* = 7.9 Hz, $H_{arom.}$), 6.76 (t, 1H, *J* = 7.9 Hz, $H_{arom.}$), 5.67 (s, 1H, O*H*), 4.45 (t, 2H, *J* = 5.4 Hz, C*H*$_2$), 3.60 (t, 2H, *J* = 5.4 Hz, C*H*$_2$), 2.94 (s, 3H, NC*H*$_3$), 1.41 (s, 9H, C(C*H*$_3$)$_3$) ppm. **$^{13}$C NMR** (101 MHz, $CDCl_3$, 50 °C): δ = 170.0 (*C*O$_2$CH$_2$), 155.6 (N*C*O$_2$C(CH$_3$)$_3$), 148.9 ($C_{arom.}$), 145.2 ($C_{arom.}$), 120.6 ($C_{arom.}$), 119.9 ($C_{arom.}$), 119.2 ($C_{arom.}$), 112.4 ($C_{arom.}$), 79.9 (O*C*(CH$_3$)$_3$), 63.2 (*C*H$_2$), 47.6 (*C*H$_2$), 35.1 (N*C*H$_3$), 28.3 (C(*C*H$_3$)$_3$) ppm. **MS** (negative ESI-FTMS, MeOH): m/z (%) = 310.12549 (100, [M−H$^+$], $C_{15}H_{20}NO_6^-$, calc. 310.12961). **IR** (in KBr): ṽ (cm$^{-1}$) = 3341, 2971, 2465, 2299, 1668, 1465, 1385, 1294, 1149, 1065, 1017, 965, 849, 736. **Elemental analysis** $C_{15}H_{21}NO_6$: calc. C 57.87 %, H 6.80 %, N 4.50 %; found C 57.81 %, H 6.77 %, N 4.46 %.

**2-((*tert*-Butoxycarbonyl)(ethyl)amino)ethyl 2,3-dihydroxybenzoate (12b):** Y = 76 % (1.23 g, 3.78 mmol, colorless solid). **$R_f$** = 0.24 (pentane /ethylacetate 8:1). **Mp** = 52 °C - 57 °C (dichloromethane). **$^1$H NMR** (400 MHz, $CDCl_3$, 50 °C): δ = 10.75 (s, 1H, O*H*), 7.34 (dd, 1H, *J* = 8.0, 1.5 Hz, $H_{arom.}$), 7.11-7.05 (m, 1H, $H_{arom.}$), 6.77 (t, 1H, *J* = 8.0 Hz, $H_{arom.}$), 5.63 (s, 1H, O*H*), 4.44 (t, 2H, *J* = 5.6 Hz, C*H*$_2$), 3.57 (t, 2H, *J* = 5.6 Hz, C*H*$_2$), 3.36-3.24 (m, 2H, NC*H*$_2$CH$_3$), 1.43 (s, 9H, C(C*H*$_3$)$_3$), 1.12 (t, 3H, *J* = 7.1 Hz, CH$_2$C*H*$_3$) ppm. **$^{13}$C NMR** (101 MHz, $CDCl_3$, 50 °C): δ = 170.0 (*C*O$_2$CH$_2$), 155.3 (N*C*O$_2$C(CH$_3$)$_3$), 148.9 ($C_{arom.}$), 145.1 ($C_{arom.}$), 120.6 ($C_{arom.}$), 119.8 ($C_{arom.}$), 119.2 ($C_{arom.}$), 112.4 ($C_{arom.}$), 79.8 (O*C*(CH$_3$)$_3$), 63.6 (*C*H$_2$), 45.6 (*C*H$_2$), 42.8 (N*C*H$_2$CH$_3$), 28.3 (C(*C*H$_3$)$_3$), 13.6 (CH$_2$*C*H$_3$) ppm. **MS** (negative ESI-FTMS, MeOH, acidified): m/z (%) = 324.14200 (100, [M−H$^+$], $C_{16}H_{22}NO_6^-$, calc. 324.14526). **IR** (in KBr): ṽ (cm$^{-1}$) = 3360, 2973, 2723, 2316, 2091, 1993, 1911, 1673, 1465, 1411, 1278, 1149, 1072, 1011, 853, 754. **Elemental analysis** $C_{16}H_{23}NO_6$: calc. C 59.07 %, H 7.13 %, N 4.31 %; found C 58.95 %, H 6.94 %, N 4.28 %.

**2-((*tert*-Butoxycarbonyl)(isopropyl)amino)ethyl 2,3-dihydroxybenzoate (12c):** Y = 65 % (1.11 g, 3.27 mmol, colorless solid). **$R_f$** = 0.21 (pentane/ethylacetate 8:1). **Mp** = 76 % - 81 % (dichloro-methane). **$^1$H NMR** (400 MHz, $CDCl_3$, 50 °C): δ = 10.80 (s, 1H, O*H*), 7.35 (dd, 1H, *J* = 8.0, 1.5 Hz, $H_{arom.}$), 7.08 (d, 1H, *J* = 8.0 Hz, $H_{arom.}$), 6.76 (t, 1H, *J* = 8.0 Hz, $H_{arom.}$), 5.67 (s, 1H, O*H*), 4.43 (t, 2H, *J* = 6.3 Hz, C*H*$_2$), 4.17 (br, 1H, C*H*(CH$_3$)$_2$), 3.46 (br, 2H, C*H*$_2$), 1.45 (s, 9H, C(C*H*$_3$)$_3$), 1.15 (d, 6H, *J* = 6.8 Hz, CH(C*H*$_3$)$_2$) ppm. **$^{13}$C NMR** (101 MHz, $CDCl_3$, 50 °C): δ = 170.1 (*C*O$_2$CH$_2$), 155.3 (N*C*O$_2$C(CH$_3$)$_3$), 148.9 ($C_{arom.}$), 145.2 ($C_{arom.}$), 120.5 ($C_{arom.}$), 119.8 ($C_{arom.}$), 119.1 ($C_{arom.}$), 112.4 ($C_{arom.}$), 79.9 (O*C*(CH$_3$)$_3$), 64.1 (*C*H$_2$), 47.7 (*C*H(CH$_3$)$_2$), 41.5 (*C*H$_2$), 28.4 (C(*C*H$_3$)$_3$), 20.8 (CH(*C*H$_3$)$_2$) ppm. **MS** (positive ESI-FTMS, MeOH): m/z (%) = 362.15637 (72, [M+Na$^+$], $C_{17}H_{25}NO_6Na^+$, calc. 362.15741). **IR** (in KBr): ṽ (cm$^{-1}$) = 3349, 3114, 2972, 2297, 2102, 1987, 1919, 1669, 1458, 1386, 1281, 1233, 1150, 1049, 957,

899, 842, 753. **Elemental analysis** $C_{17}H_{25}NO_6$: calc. C 60.16 %, H 7.43 %, N 4.13 %; found C 59.86 %, H 7.26 %, N 4.47 %.

***tert*-Butyl-4-((2,3-dihydroxybenzoyl)oxy)piperidine-1-carboxylate (12d):** Y = 48 % (813 mg, 2.41 mmol, colorless solid). **$R_f$** = 0.27 (pentane /ethylacetate 6:1). **Mp** = 103 °C - 109 °C (dichloromethane). **$^1$H NMR** (600 MHz, $CDCl_3$, 25 °C): δ = 10.89 (s, 1H, O*H*), 7.31 (dd, 1H, *J* = 8.0, 1.4 Hz, $H_{arom.}$), 7.09-7.05 (m, 1H, $H_{arom.}$), 6.73 (t, 1H, *J* = 8.0 Hz, $H_{arom.}$), 6.35 (s, 1H, O*H*), 5.24-5.15 (m, 1H, $CO_2CH$), 3.69 (br, 2H, $H_{cycl.}$), 3.39-3.31 (m, 2H, $H_{cycl.}$), 1.96-1.89 (m, 2H, $H_{cycl.}$), 1.78-1.71 (m, 2H, $H_{cycl.}$), 1.44 (s, 9H, $C(CH_3)_3$) ppm. **$^{13}$C NMR** (151 MHz, $CDCl_3$, 25 °C): δ = 169.6 ($CO_2CH$), 154.8 ($NCO_2C(CH_3)_3$), 149.3 ($C_{arom.}$), 145.2 ($C_{arom.}$), 120.3 ($C_{arom.}$), 120.2 ($C_{arom.}$), 119.1 ($C_{arom.}$), 112.6 ($C_{arom.}$), 79.9 ($OC(CH_3)_3$), 71.0 ($CO_2CH$), 41.1 ($C_{cycl.}$), 40.4 ($C_{cycl.}$), 30.4 (2×$C_{cycl.}$), 28.4 ($C(CH_3)_3$) ppm. **MS** (negative ESI-FTMS, MeOH): m/z (%) = 336.14859 (100, [M−H$^+$], $C_{17}H_{22}NO_6^-$, calc. 336.14526). **IR** (in KBr): ṽ (cm$^{-1}$) = 3377, 3094, 2961, 2306, 2100, 1668, 1443, 1267, 1143, 1012, 850, 749. **Elemental analysis** $C_{17}H_{23}NO_6$ × 0.5 $H_2O$: calc. C 58.95 %, H 6.98 %, N 4.04 %; found C 58.59 %, H 6.47 %, N 4.07 %.

**2-(Methylamino)ethyl 2,3-dihydroxybenzoate hydrochloride (13a-$H_2$):** Y = 93 % (230 mg, 0.93 mmol, colorless solid). **Mp** = 234 °C - 237 °C (last solvent: glacial acetic acid). **$^1$H NMR** (600 MHz, DMSO-$d_6$, 25 °C): δ = 10.24 (s, 1H, O*H*), 9.53 (s, 1H, O*H*), 9.19 (s, 2H, $NH_2$), 7.46 (dd, 1H, *J* = 7.9, 1.5 Hz, $H_{arom.}$), 7.08 (dd, 1H, *J* = 7.9, 1.5 Hz, $H_{arom.}$), 6.76 (t, 1H, *J* = 7.9 Hz, $H_{arom.}$), 4.60-4.53 (m, 2H, $CH_2$), 3.33 (br, 2H, $CH_2$), 2.61 (s, 3H, $NCH_3$) ppm. **$^{13}$C NMR** (151 MHz, DMSO-$d_6$, 25 °C): δ = 169.4 ($CO_2CH_2$), 149.8 ($C_{arom.}$), 146.5 ($C_{arom.}$), 121.4 ($C_{arom.}$), 120.9 ($C_{arom.}$), 119.2 ($C_{arom.}$), 113.4 ($C_{arom.}$), 61.2 ($CH_2$), 47.2 ($CH_2$), 33.1 ($NCH_3$) ppm. **MS** (positive ESI-FTMS, MeOH): m/z (%) = 212.09157 (100, [M−Cl$^-$], $C_{10}H_{14}NO_4^+$, calc. 212.09173). **IR** (in KBr): ṽ (cm$^{-1}$) = 3362, 3036, 2956, 2731, 2465, 2295, 2183, 2059, 1987, 1867, 1675, 1606, 1465, 1386, 1276, 1141, 1069, 1019, 968, 900, 847, 751, 700. **Elemental analysis** $C_{10}H_{14}NO_4Cl$: calc. C 48.50 %, H 5.70 %, N 5.66 %; found C 48.62 %, H 5.89 %, N 5.71 %.

**2-(Ethylamino)ethyl 2,3-dihydroxybenzoate hydrochloride (13b-$H_2$):** Y = quantitative (263 mg, 1.01 mmol, colorless solid). **Mp** = decomposition above 197 °C. **$^1$H NMR** (600 MHz, DMSO-$d_6$, 25 °C): δ = 10.24 (s, 1H, O*H*), 9.54 (s, 1H, O*H*), 9.25 (s, 2H, $NH_2$), 7.46 (dd, 1H, *J* = 7.9, 1.5 Hz, $H_{arom.}$), 7.08 (dd, 1H, *J* = 7.9, 1.5 Hz, $H_{arom.}$), 6.76 (t, 1H, *J* = 7.9 Hz, $H_{arom.}$), 4.60-4.54 (m, 2H, $CH_2$), 3.35-3.30 (m, 2H, $CH_2$), 3.06-2.98 (m, 2H, $NCH_2CH_3$), 1.23 (t, 3H, *J* = 7.3 Hz, $CH_2CH_3$) ppm. **$^{13}$C NMR** (151 MHz, DMSO-$d_6$, 25 °C): δ = 169.4 ($CO_2CH_2$), 149.8 ($C_{arom.}$), 146.5 ($C_{arom.}$), 121.3 ($C_{arom.}$), 120.9 ($C_{arom.}$), 119.2 ($C_{arom.}$), 113.5 ($C_{arom.}$), 61.3 ($CH_2$), 45.2 ($CH_2$), 42.7 ($NCH_2CH_3$), 11.3 ($CH_2CH_3$) ppm. **MS** (positive ESI-FTMS, MeOH): m/z (%) = 226.10776 (100, [M−Cl$^-$], $C_{11}H_{16}NO_4^+$, calc. 226.10738). **IR** (in KBr): ṽ (cm$^{-1}$) = 3346, 3202, 3058, 2957, 2724, 2489, 2401, 2261, 2099, 1925, 1816, 1676, 1595, 1469, 1409, 1363, 1267, 1142, 1066, 1024, 967, 907, 847, 805, 750, 696. **Elemental analysis** $C_{11}H_{16}NO_4Cl$ × 0.25 $H_2O$: calc. C 49.63 %, H 6.25 %, N 5.26 %; found C 49.70 %, H 6.21 %, N 5.40 %.

**2-(Isopropylamino)ethyl 2,3-dihydroxybenzoate hydrochloride (13c-$H_2$):** Y = 61 % (168 mg, 0.61 mmol, colorless solid). **Mp** = decomposition above 212 °C. **$^1$H NMR** (600 MHz, DMSO-$d_6$, 25 °C): δ = 10.22 (s, 1H, O*H*), 9.52 (s, 1H, O*H*), 9.19 (s, 2H, $NH_2$), 7.42 (dd, 1H, *J* = 7.9, 1.4 Hz, $H_{arom.}$), 7.06 (dd, 1H, *J* = 7.9, 1.4 Hz, $H_{arom.}$), 6.75 (t, 1H, *J* = 7.9 Hz, $H_{arom.}$), 4.60-4.52 (m, 2H, $CH_2$), 3.39-3.28 (m, 3H, $CH_2$ and

C*H*($CH_3$)$_2$), 1.26 (d, 6H, *J* = 6.5 Hz, CH(C*H*$_3$)$_2$) ppm. **$^{13}$C NMR** (151 MHz, DMSO-*d*$_6$, 25 °C): δ = 169.4 (*C*$O_2CH_2$), 149.8 ($C_{arom.}$), 146.5 ($C_{arom.}$), 121.3 ($C_{arom.}$), 120.8 ($C_{arom.}$), 119.2 ($C_{arom.}$), 113.5 ($C_{arom.}$), 61.4 (*C*$H_2$), 50.3 (*C*H($CH_3$)$_2$), 42.9 (*C*$H_2$), 18.9 (CH(*C*$H_3$)$_2$) ppm. **MS** (positive ESI-FTMS, MeOH, acidified): m/z (%) = 240.12228 (100, [M−Cl$^-$], $C_{12}H_{18}NO_4^+$, calc. 240.12303). **IR** (in KBr): $\tilde{\nu}$ (cm$^{-1}$) = 3780, 3610, 3276, 2976, 2779, 2686, 2505, 2449, 2342, 2190, 2121, 2077, 2012, 1918, 1881, 1664, 1609, 1556, 1460, 1350, 1302, 1260, 1143, 1065, 1024, 960, 894, 848, 787, 719. **Elemental analysis** $C_{12}H_{18}NO_4Cl$ × 0.25 $H_2O$: calc. C 51.43 %, H 6.65 %, N 5.00 %; found C 51.45 %, H 6.62 %, N 4.87 %.

**Piperidin-4-yl 2,3-dihydroxybenzoate hydrochloride (13d-H$_2$):** Y = 57 % (93.1 mg, 0.34 mmol, colorless solid). **Mp** = decomposition above 277 °C. **$^1$H NMR** (600 MHz, DMSO-*d*$_6$, 25 °C): δ = 10.30 (s, 1H, O*H*), 9.51 (s, 1H, O*H*), 9.02 (s, 2H, N*H*$_2$), 7.30 (dd, 1H, *J* = 7.9, 1.4 Hz, $H_{arom.}$), 7.05 (dd, 1H, *J* = 7.9, 1.4 Hz, $H_{arom.}$), 6.75 (t, 1H, *J* = 7.9 Hz, $H_{arom.}$), 5.25-5.18 (m, 1H, $CO_2$C*H*), 3.28-3.18 (m, 2H, $H_{cycl.}$), 3.16-3.08 (m, 2H, $H_{cycl.}$), 2.16-2.07 (m, 2H, $H_{cycl.}$), 1.98-1.91 (m, 2H, $H_{cycl.}$) ppm. **$^{13}$C NMR** (151 MHz, DMSO-*d*$_6$, 25 °C): δ = 168.9 (*C*$O_2$CH), 149.9 ($C_{arom.}$), 146.6 ($C_{arom.}$), 121.2 ($C_{arom.}$), 120.3 ($C_{arom.}$), 119.4 ($C_{arom.}$), 113.8 ($C_{arom.}$), 68.0 ($CO_2$*C*H), 40.6 (2×$C_{cycl.}$), 27.3 (2×$C_{cycl.}$) ppm. **MS** (positive ESI-FTMS, MeOH, acidified): m/z (%) = 238.10698 (100, [M−Cl$^-$], $C_{12}H_{16}NO_4^+$, calc. 238.10738). **IR** (in KBr): $\tilde{\nu}$ (cm$^{-1}$) = 3280, 3206, 2942, 2786, 2753, 2548, 2512, 2456, 2328, 2196, 2165, 2079, 1991, 1952, 1868, 1677, 1593, 1533, 1464, 1374, 1345, 1312, 1252, 1217, 1141, 1065, 1023, 981, 937, 907, 844, 817, 782, 750, 689. **Elemental analysis** $C_{12}H_{16}NO_4Cl$: calc. C 52.66 %, H 5.89 %, N 5.12 %; found C 52.61 %, H 5.89 %, N 5.09 %.

**$Li_4$[(13a)$_1$(2)$_5$$Ti_2$]:** Y = quantitative (266.6 mg, red solid). **MS** (negative ESI-FTMS, THF): m/z (%) = 1559.41724 (3, [$Li_3$[(**13a**)$_2$(**2**)$_4$$Ti_2$]]$^-$, $C_{80}H_{70}N_2O_{24}Li_3Ti_2^-$, calc. 1559.37631), 1606.42517 (28, [$Li_3$[(**13a**)$_1$(**2**)$_5$$Ti_2$]]$^-$, $C_{85}H_{71}NO_{24}Li_3Ti_2^-$, calc. 1606.38106), 1653.42896 (100, [$Li_3$[(**2**)$_6$$Ti_2$]]$^-$, $C_{90}H_{72}O_{24}Li_3Ti_2^-$, calc. 1653.38581).

**$Li_4$[(13b)$_1$(2)$_5$$Ti_2$]:** Y = quantitative (332.9 mg, red solid). **MS** (negative ESI-FTMS, THF): m/z (%) = 1587.45703 (4, [$Li_3$[(**13b**)$_2$(**2**)$_4$$Ti_2$]]$^-$, $C_{82}H_{74}N_2O_{24}Li_3Ti_2^-$, calc. 1587.40761), 1620.44763 (52, [$Li_3$[(**13b**)$_1$(**2**)$_5$$Ti_2$]]$^-$, $C_{86}H_{73}NO_{24}Li_3Ti_2^-$, calc. 1620.39671), 1653.43530 (100, [$Li_3$[(**2**)$_6$$Ti_2$]]$^-$, $C_{90}H_{72}O_{24}Li_3Ti_2^-$, calc. 1653.38581).

**$Li_4$[(13c)$_1$(2)$_5$$Ti_2$]:** Y = quantitative (335.7 mg, red solid). **MS** (negative ESI-FTMS, THF): m/z (%) = 1615.48438 (5, [$Li_3$[(**13c**)$_2$(**2**)$_4$$Ti_2$]]$^-$, $C_{84}H_{78}N_2O_{24}Li_3Ti_2^-$, calc. 1615.43891), 1634.45715 (40, [$Li_3$[(**13c**)$_1$(**2**)$_5$$Ti_2$]]$^-$, $C_{87}H_{75}NO_{24}Li_3Ti_2^-$, calc. 1634.41236), 1653.42725 (100, [$Li_3$[(**2**)$_6$$Ti_2$]]$^-$, $C_{90}H_{72}O_{24}Li_3Ti_2^-$, calc. 1653.38581).

**$Li_4$[(13d)$_1$(2)$_5$$Ti_2$]:** Y = quantitative (335.3 mg, red solid). **MS** (negative ESI-FTMS, THF): m/z (%) = 1632.44849 (5, [$Li_3$[(**13d**)$_1$(**2**)$_5$$Ti_2$]]$^-$, $C_{87}H_{73}NO_{24}Li_3Ti_2^-$, calc. 1632.39671), 1653.43445 (100, [$Li_3$[(**2**)$_6$$Ti_2$]]$^-$, $C_{90}H_{72}O_{24}Li_3Ti_2^-$, calc. 1653.38581).

**$Li_4$[(13b)$_1$(4)$_5$$Ti_2$]:** Y = quantitative (241.2 mg, red solid). **MS** (negative ESI-FTMS, THF): m/z (%) = 1680.45349 (51, [$Li_3$[(**13b**)$_1$(**4**)$_5$$Ti_2$]]$^-$, $C_{91}H_{73}NO_{24}Li_3Ti_2^-$, calc. 1680.39671), 1725.44128 (100, [$Li_3$[(**4**)$_6$$Ti_2$]]$^-$, $C_{96}H_{72}O_{24}Li_3Ti_2^-$, calc. 1725.38581).

**$Li_4$[(13b)$_1$(5)$_5$$Ti_2$]:** Y = quantitative (155.5 mg, red solid). **MS** (negative ESI-FTMS, THF): m/z (%) = 1780.75793 (11, [$Li_3$[(**13b**)$_1$(**5**)$_5$$Ti_2$]]$^-$, $C_{96}H_{113}NO_{24}Li_3Ti_2^-$, calc. 1780.70971), 1845.80933 (100, [$Li_3$[(**5**)$_6$$Ti_2$]]$^-$, $C_{102}H_{120}O_{24}Li_3Ti_2^-$, calc. 1845.76141).

**Separation of enantio-/diastereomers:** Diels–Alder products were separated using supercritical fluid chromatography (Thar SFC). **9a:** IA column, 5–40% MeOH/$CO_2$, gradient, 35 min, 4.0 mL/min, $t_{ret}$ (major) = 14.1 min, $t_{ret}$ (minor) = 20.1 min; **9b:** IA column, 35% MeOH/$CO_2$, 4.0 mL/min, $t_{ret}$ (major) = 3.9 min, $t_{ret}$ (minor) = 9.2 min; **9d:** OJ-H column, 8–30% MeOH/$CO_2$, gradient, 35 min, 4.0 mL/min, $t_{ret}$ (major) = 7.2 min, $t_{ret}$ (minor) = 12.3 min; **9e:** IA column, 5–40% MeOH/$CO_2$, gradient, 35 min, 4.0 mL/min, $t_{ret}$ (major) = 16.7 min, $t_{ret}$ (minor) = 23.7 min. Diels–Alder product **9c** was separated by chiral HPLC: IA column, *n*-heptane/isopropyl alcohol 95:5, gradient, 40 min, 1.0 mL/min, $t_{ret}$ (major) = 25.0 min, $t_{ret}$ (minor) = 29.8 min. Nitro-Michael product **16** was separated by chiral HPLC. **16:**[9] OD-H column, *n*-heptane/isopropyl alcohol 75:25, 0.8 mL/min, $t_{ret}$ (major diastereomer pair) = 16.6 min and 23.4 min, $t_{ret}$ (minor diastereomer pair) = 20.5 min and 26.8 min.

## Possible isomers in a statistical mixture[1]

The curve and bars in the picture are only for the visualization of an ideal statistical distribution. Reactive diene ligands are colored in pink and chiral ligands are colored in green. The amount of each complex is not determined quantitatively. Only isomers in which one chiral and one reactive ligand are opposite to each other can result in an enantiomerically enriched product. Only 9 isomers of 13 follow this rule.

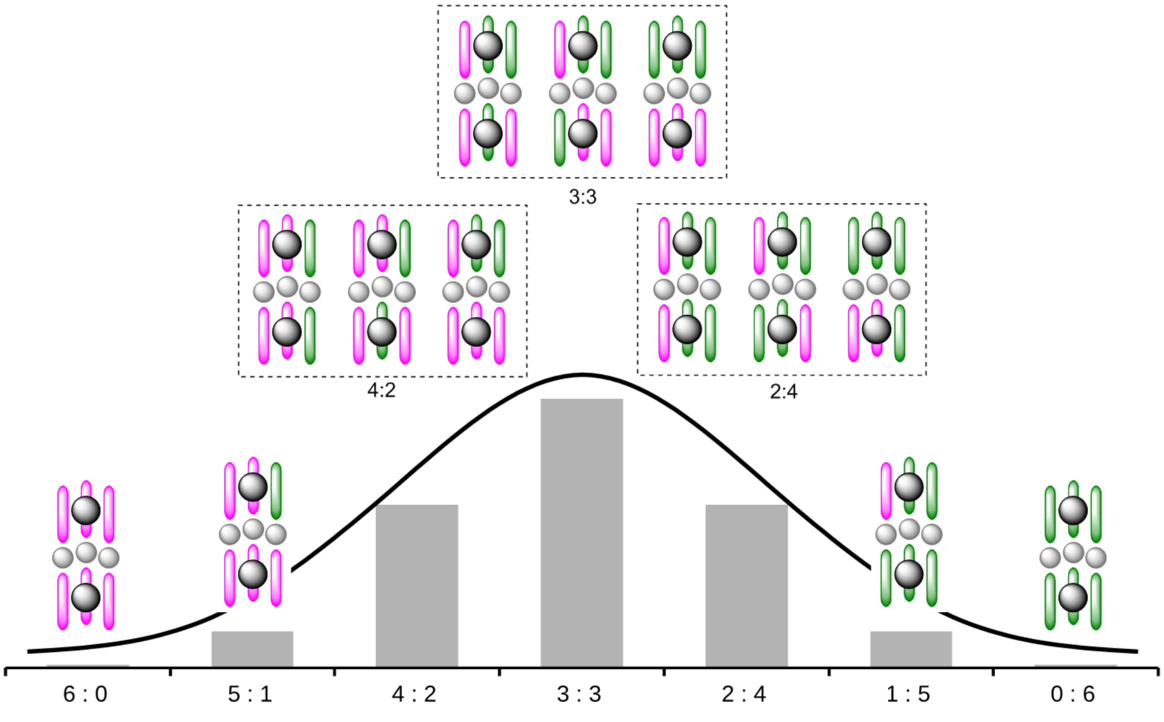

## ESI-MS Spectra of statistical helicates

**$Li_4[(1)_3(2)_3Ti_2]$:**[1]

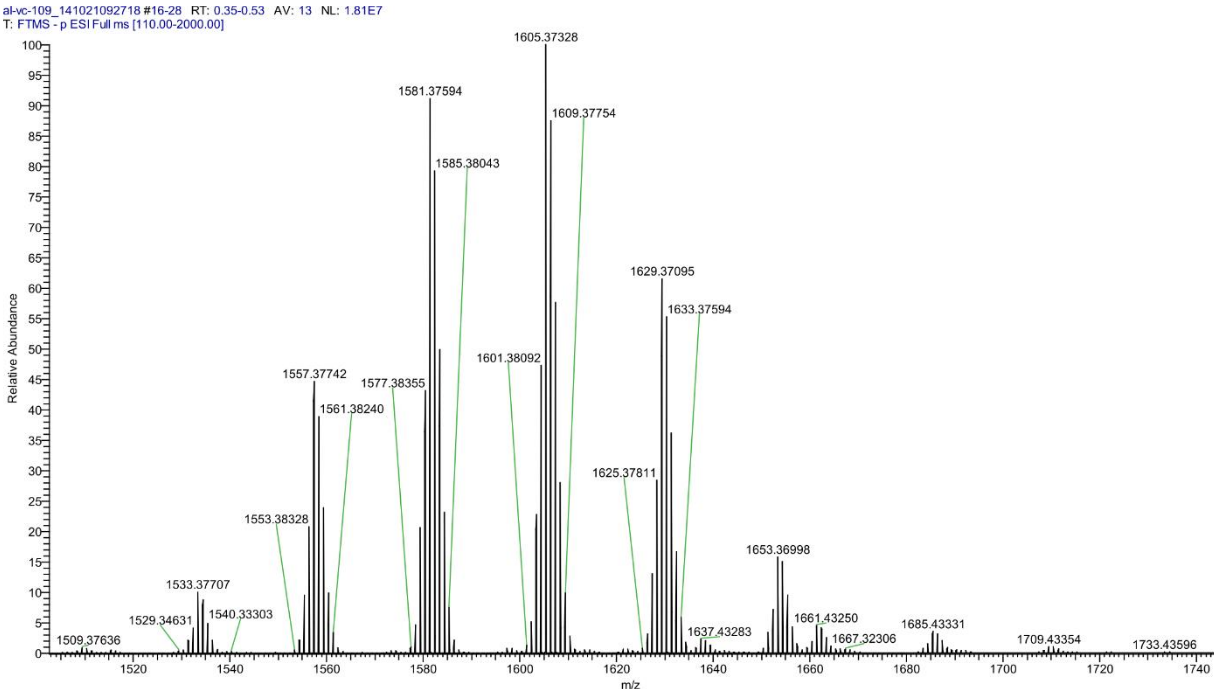


**$Li_4[(1)_3(3)_3Ti_2]$:**

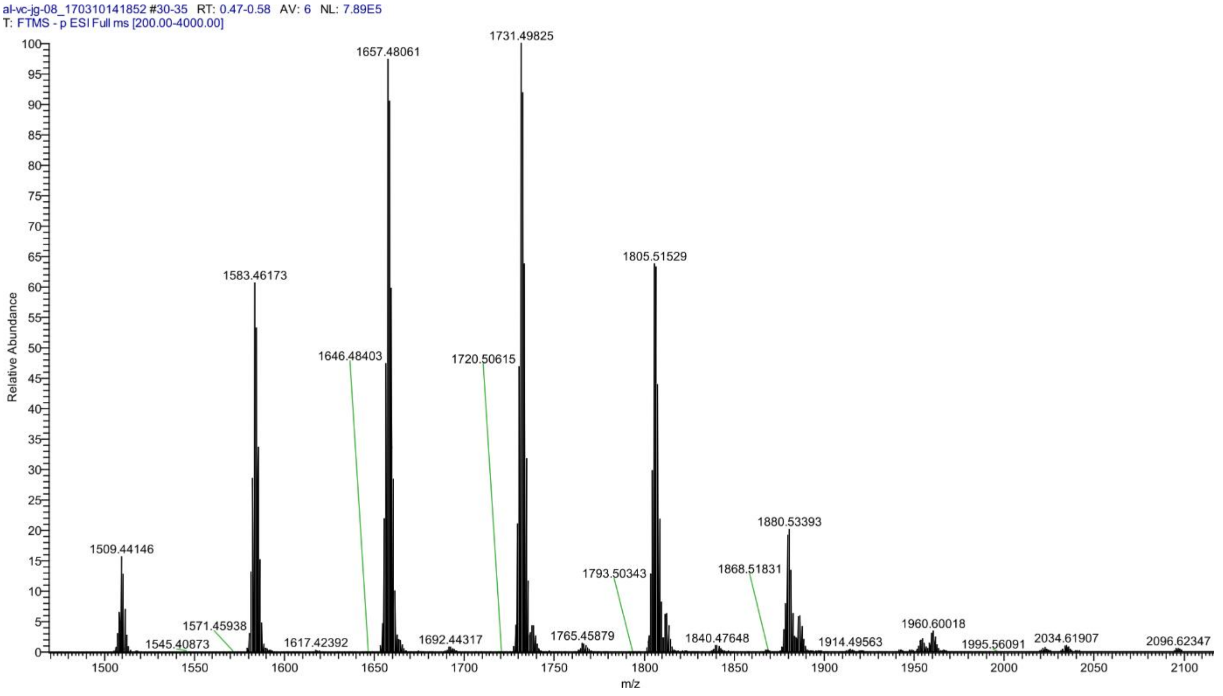

**Li$_4$[(1)$_3$(4)$_3$Ti$_2$]:**

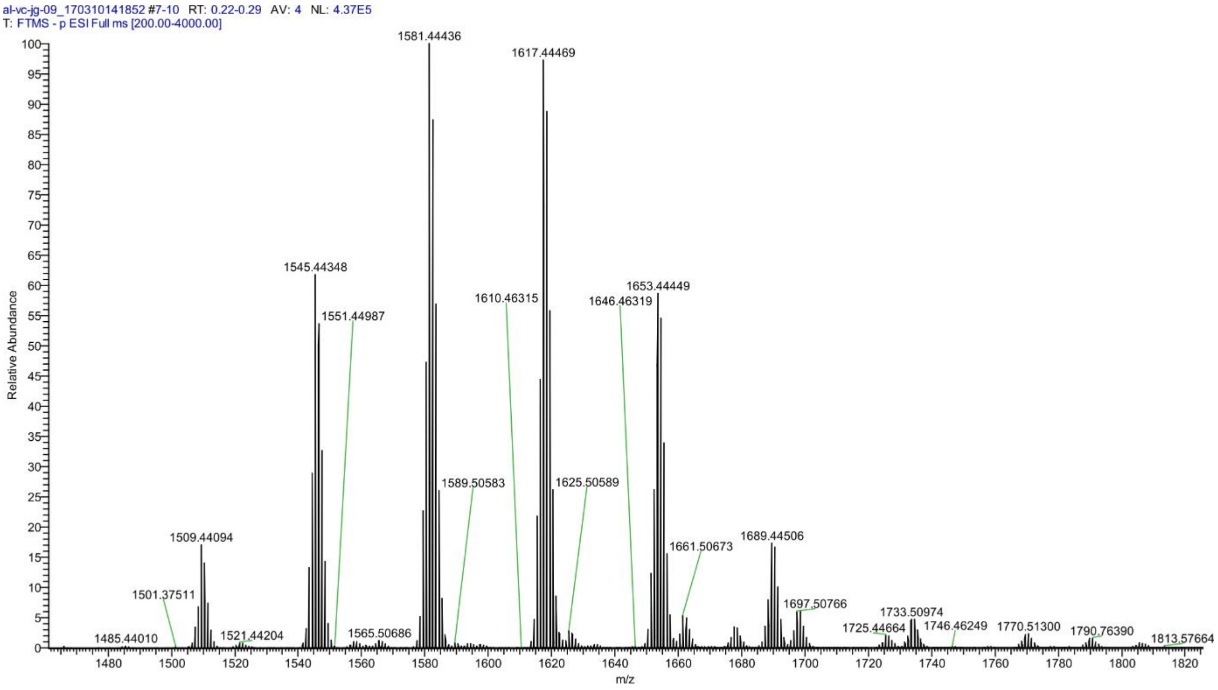


**Li$_4$[(1)$_3$(5)$_3$Ti$_2$]:**

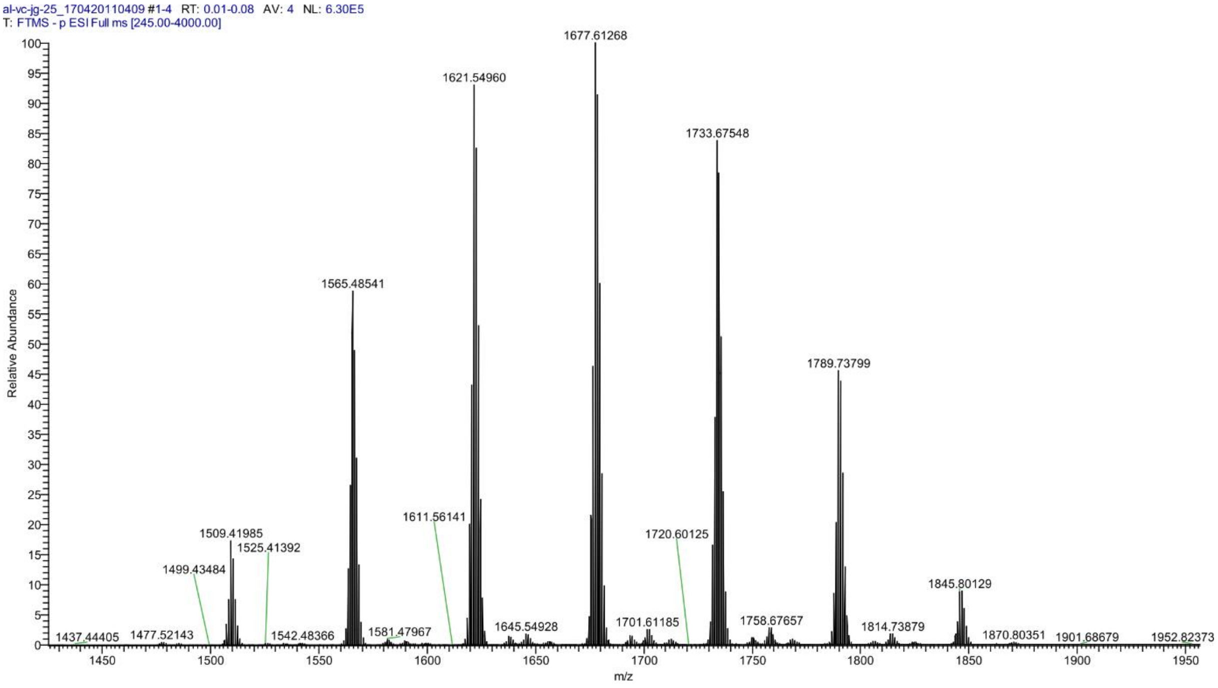

**Li$_4$[(1)$_3$(6)$_3$Ti$_2$]:**

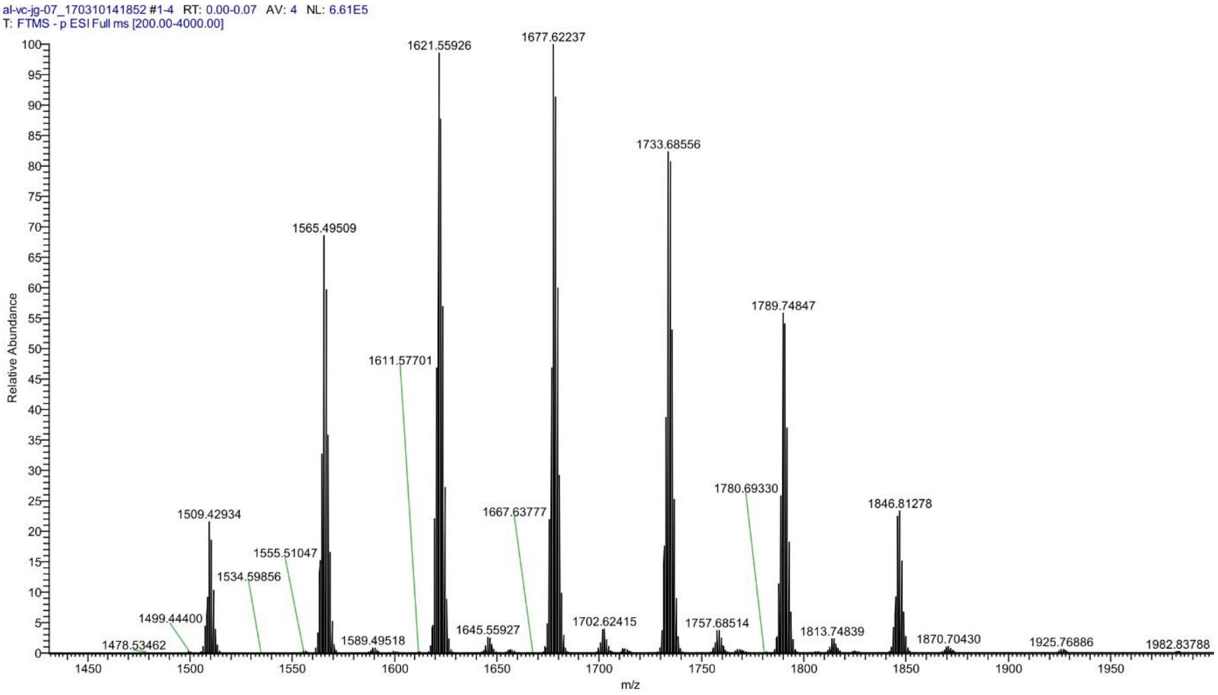


**Li$_4$[(1)$_3$(7)$_3$Ti$_2$]:**

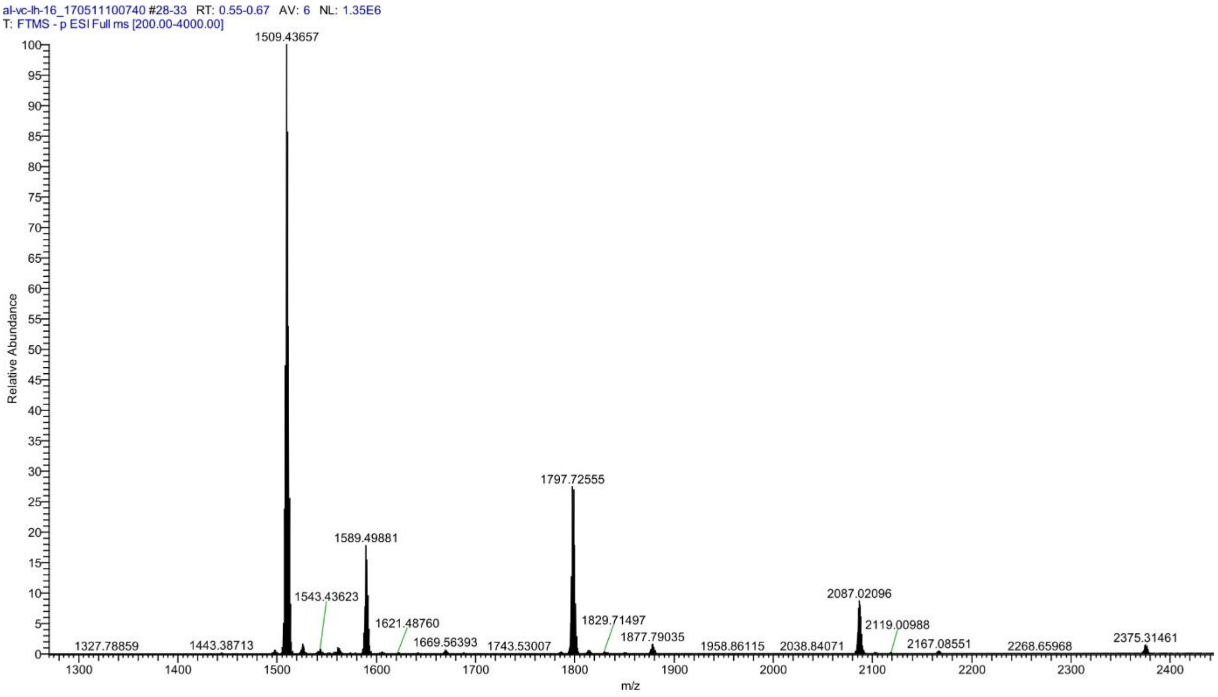

**CD spectrum of $Li_4[(4)_6Ti_2]$[10] and $Li_4[(7)_6Ti_2]$**

**$Li_4[(4)_6Ti_2]$:** The dimer is the major species in methanol with a dimerization constant of 23889 $M^{-1}$. The following CD spectrum was measured in methanol.

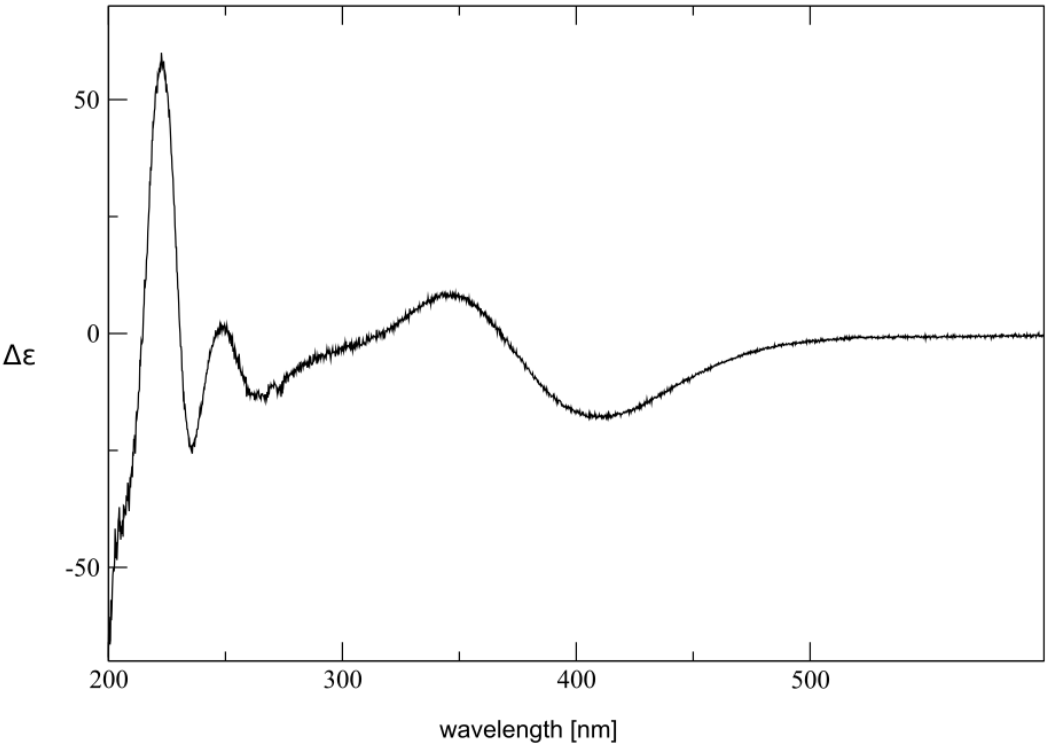


**$Li_4[(7)_6Ti_2]$:** Only dimer is observed in THF.

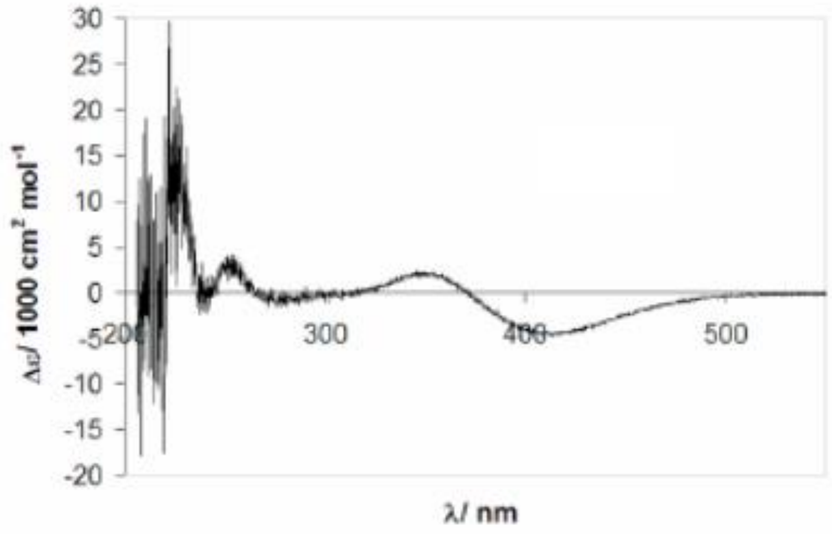

**(*S*)-1-Indanyl 2,3-dihydroxybenzoate (4-$H_2$):**

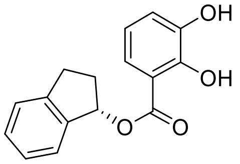


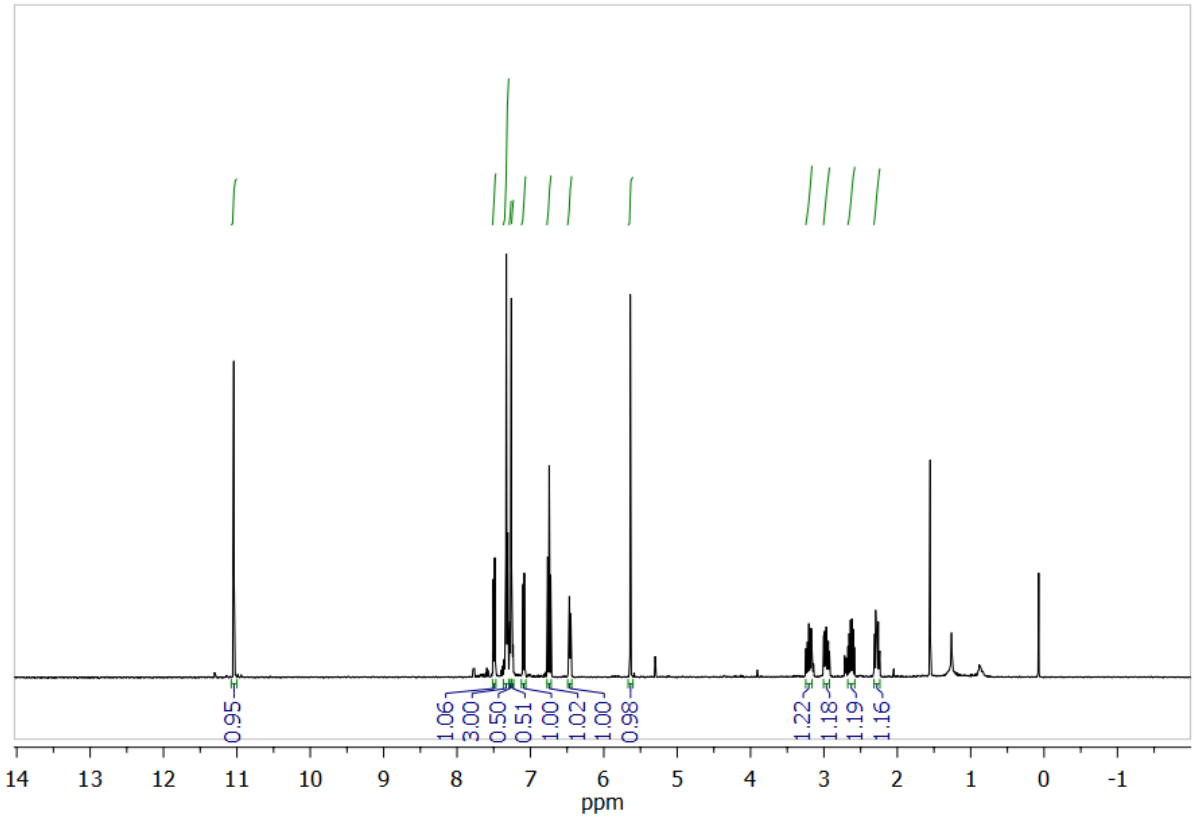


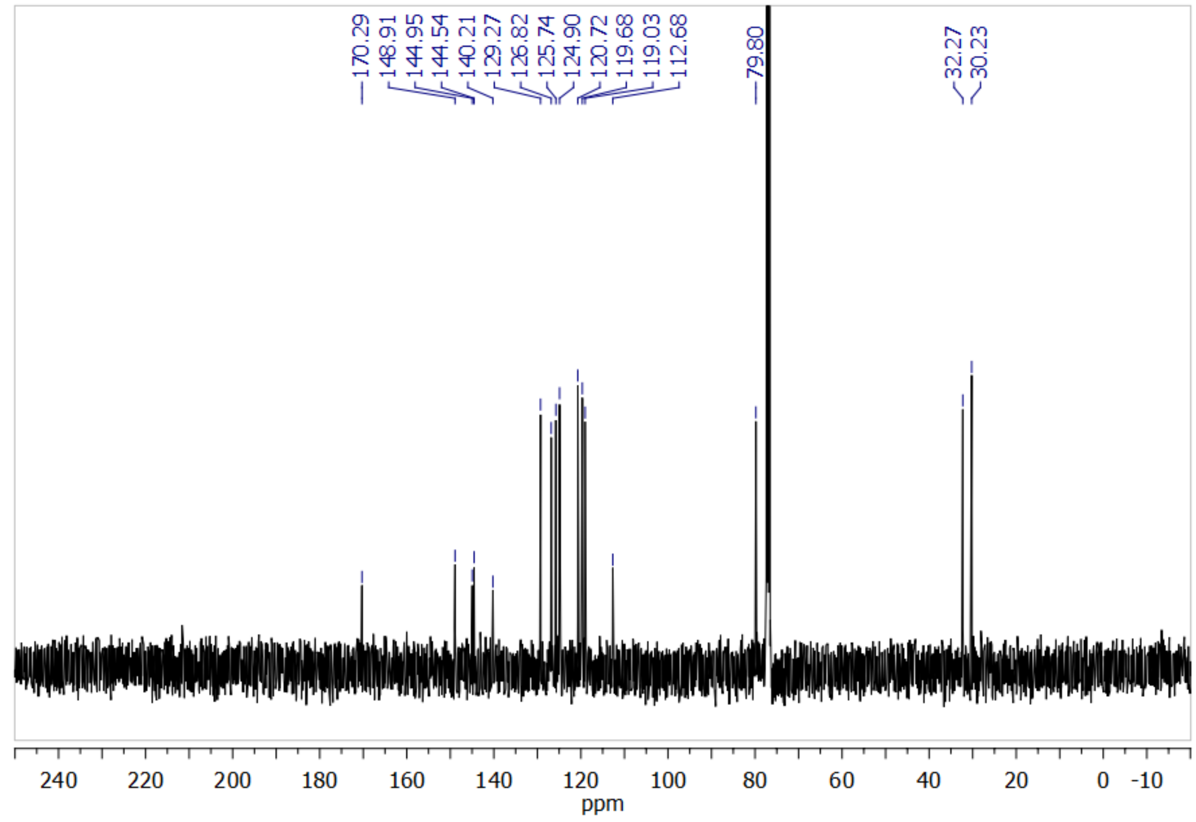

**Cholesteryl 2,3-dihydroxybenzoate (7-$H_2$):**

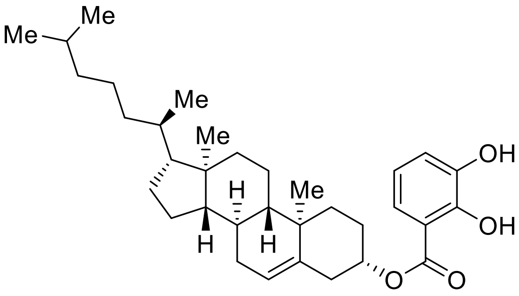


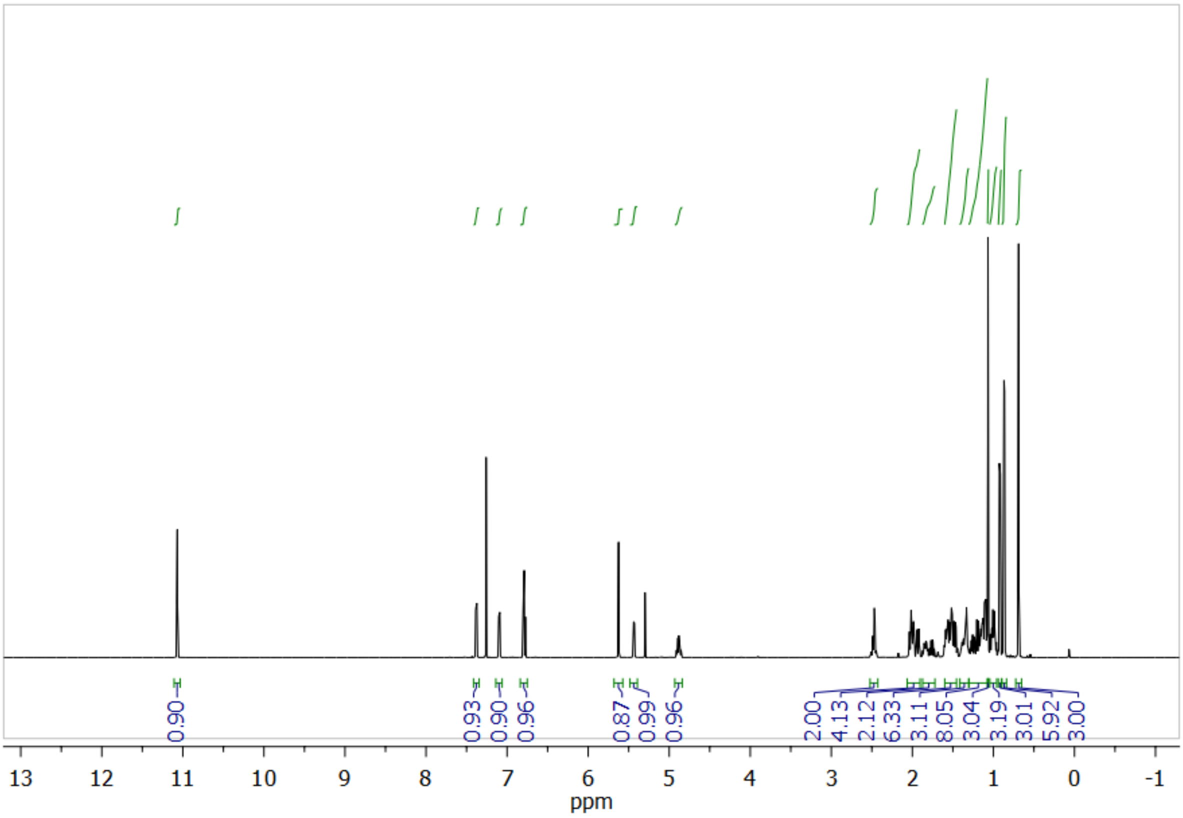


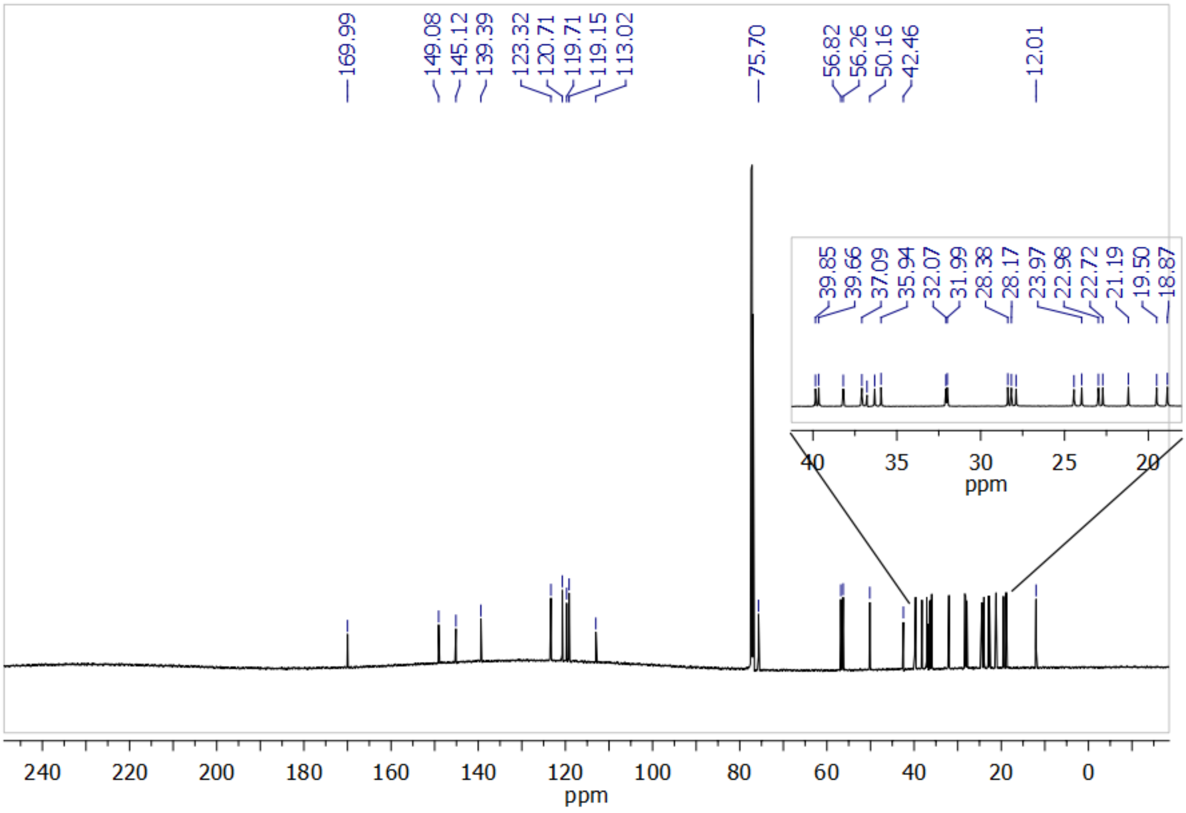

***tert*-Butyl (2-hydroxyethyl)(isopropyl)carbamate (11c):**

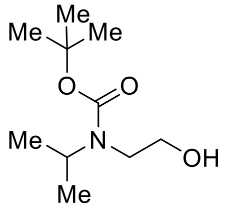


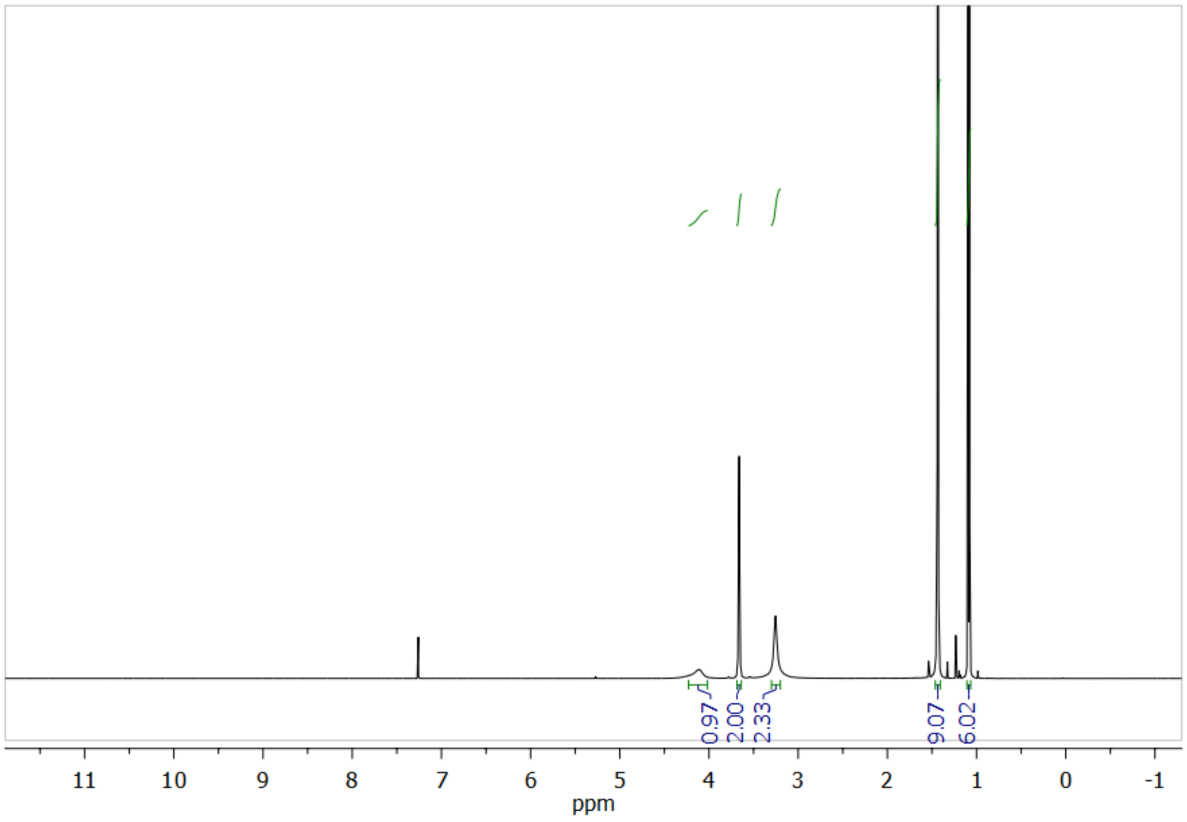


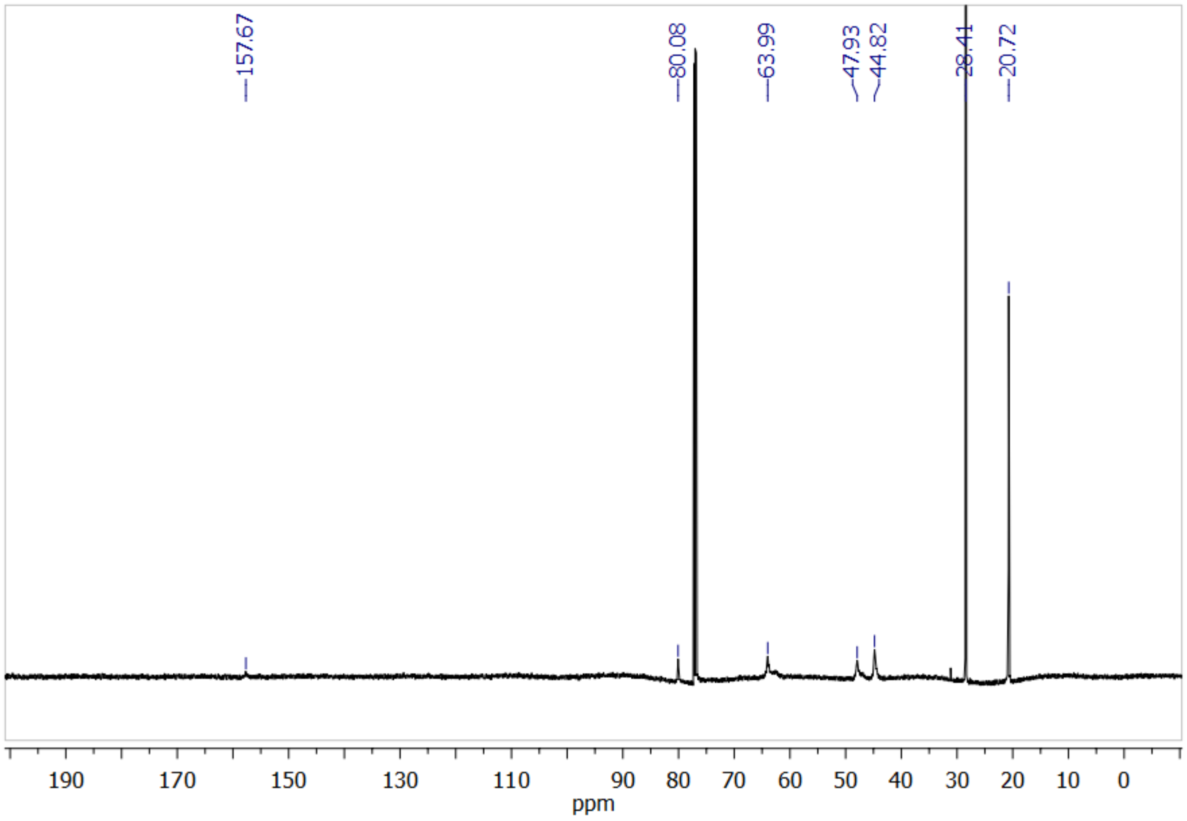

***tert*-Butyl 4-hydroxypiperidine-1-carboxylate (11d):**

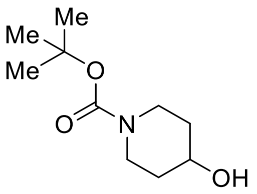


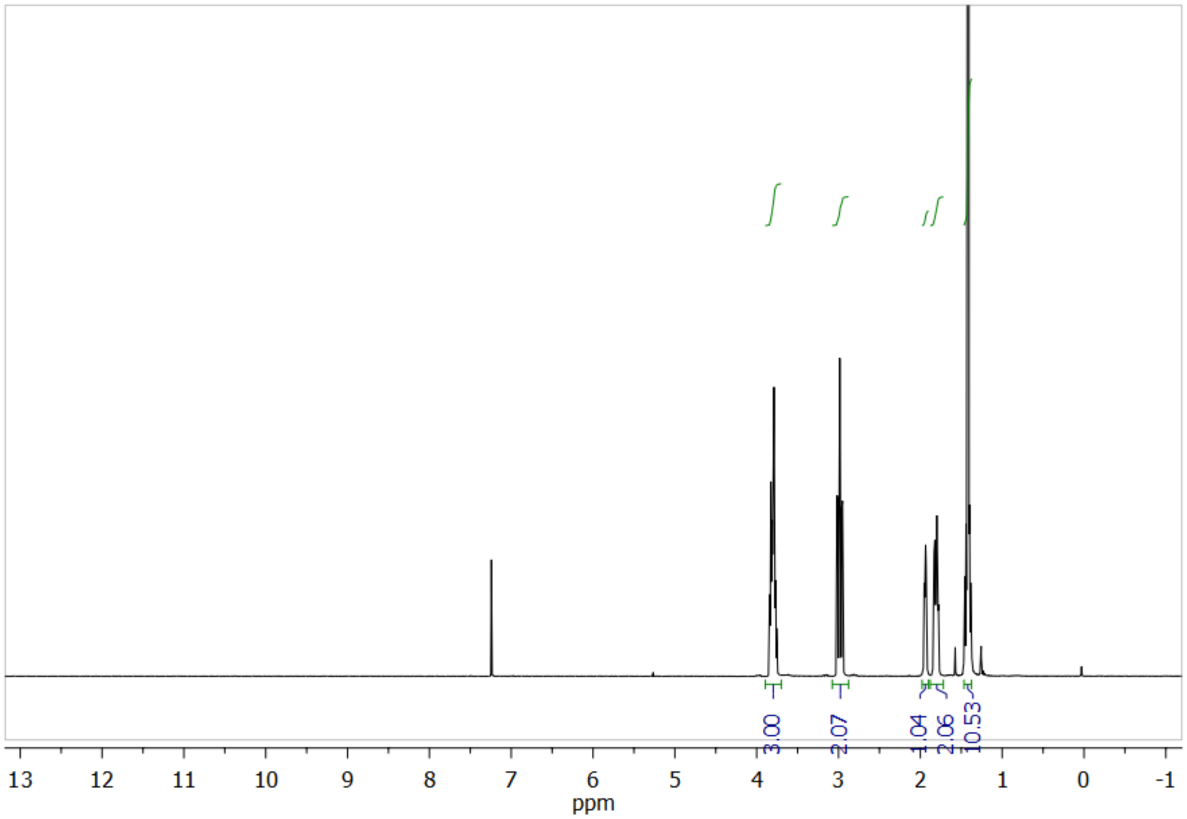


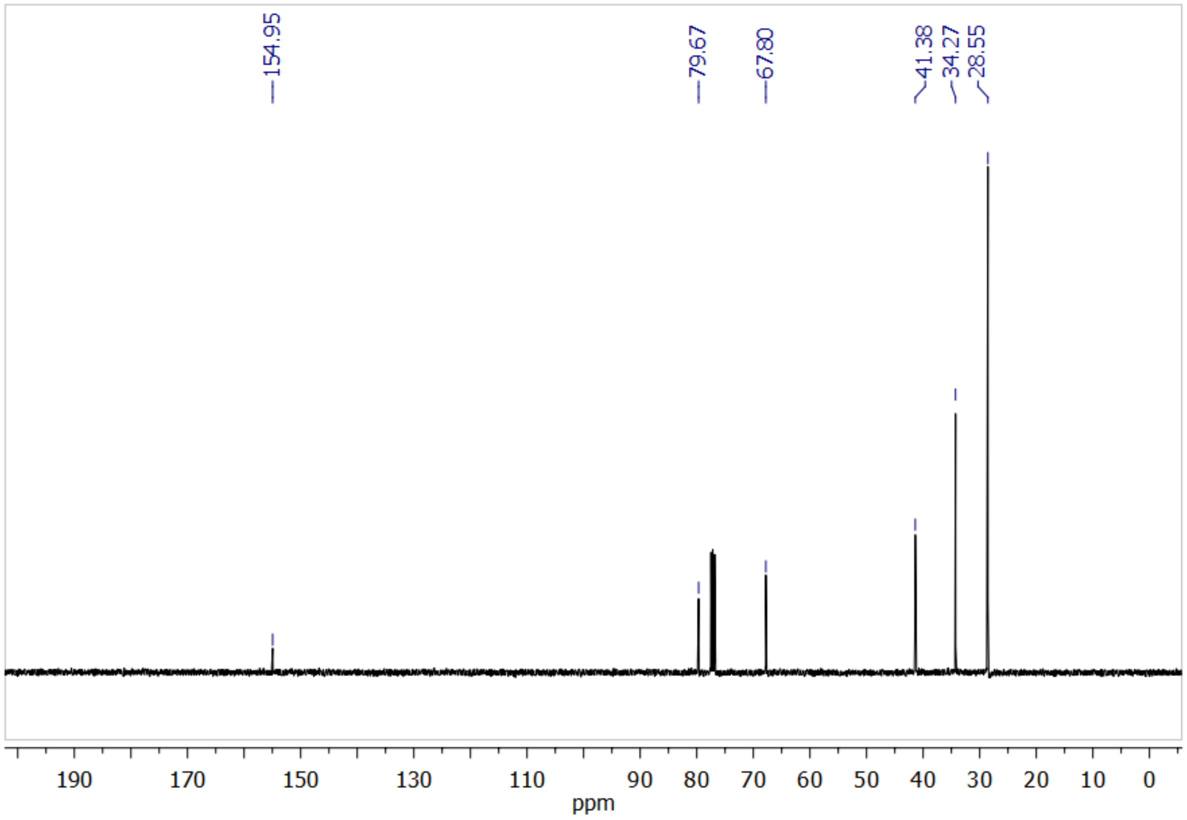

**2-((*tert*-Butoxycarbonyl)(methyl)amino)ethyl 2,3-dihydroxybenzoate (12a):**

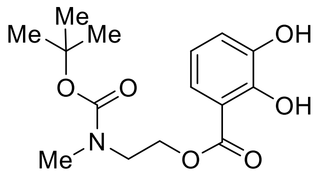


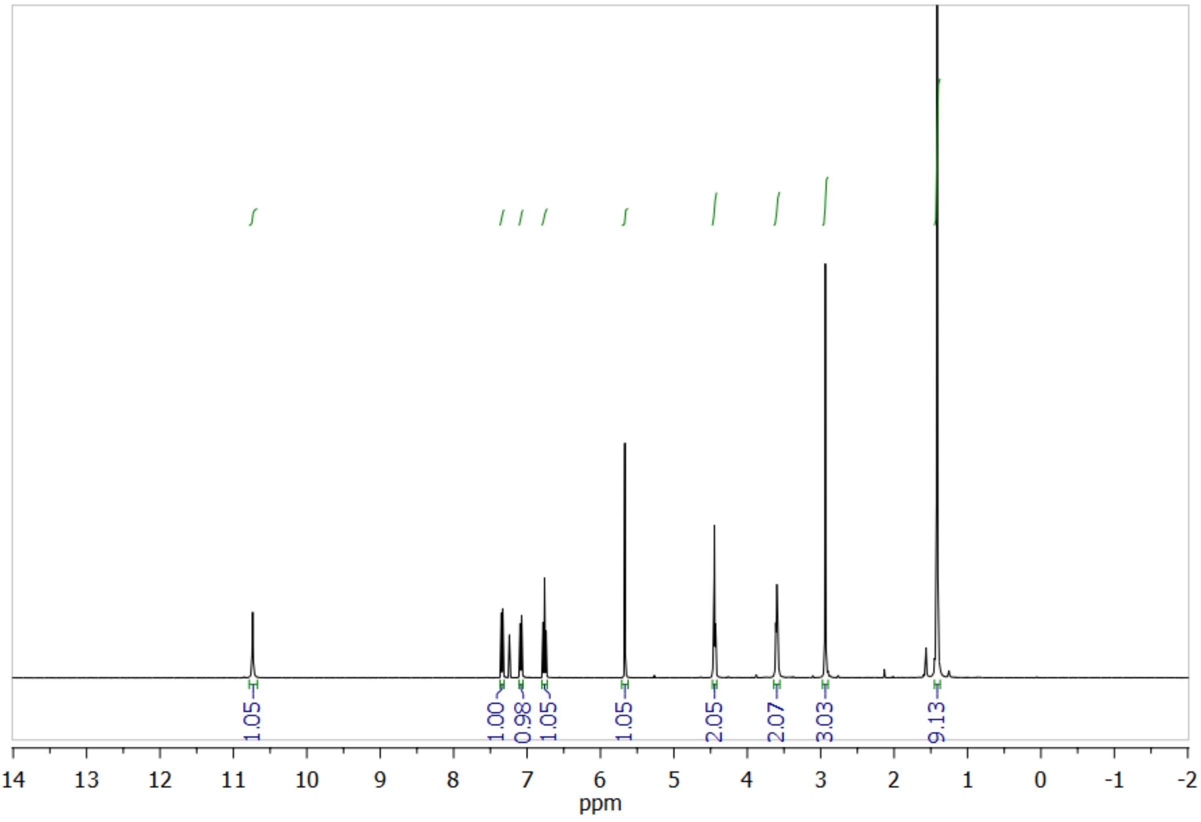


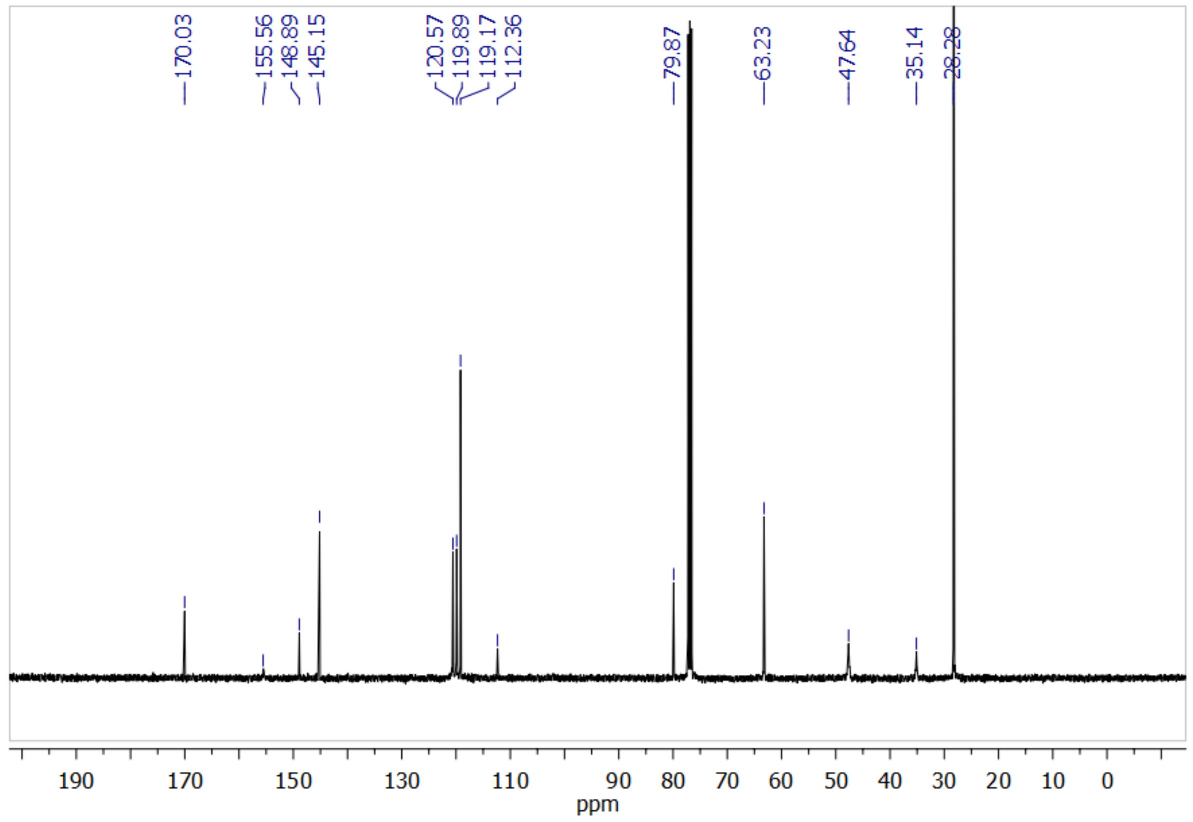

**2-((*tert*-Butoxycarbonyl)(ethyl)amino)ethyl 2,3-dihydroxybenzoate (12b):**

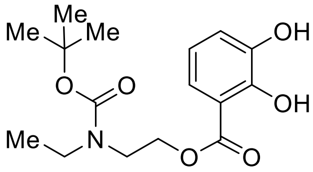


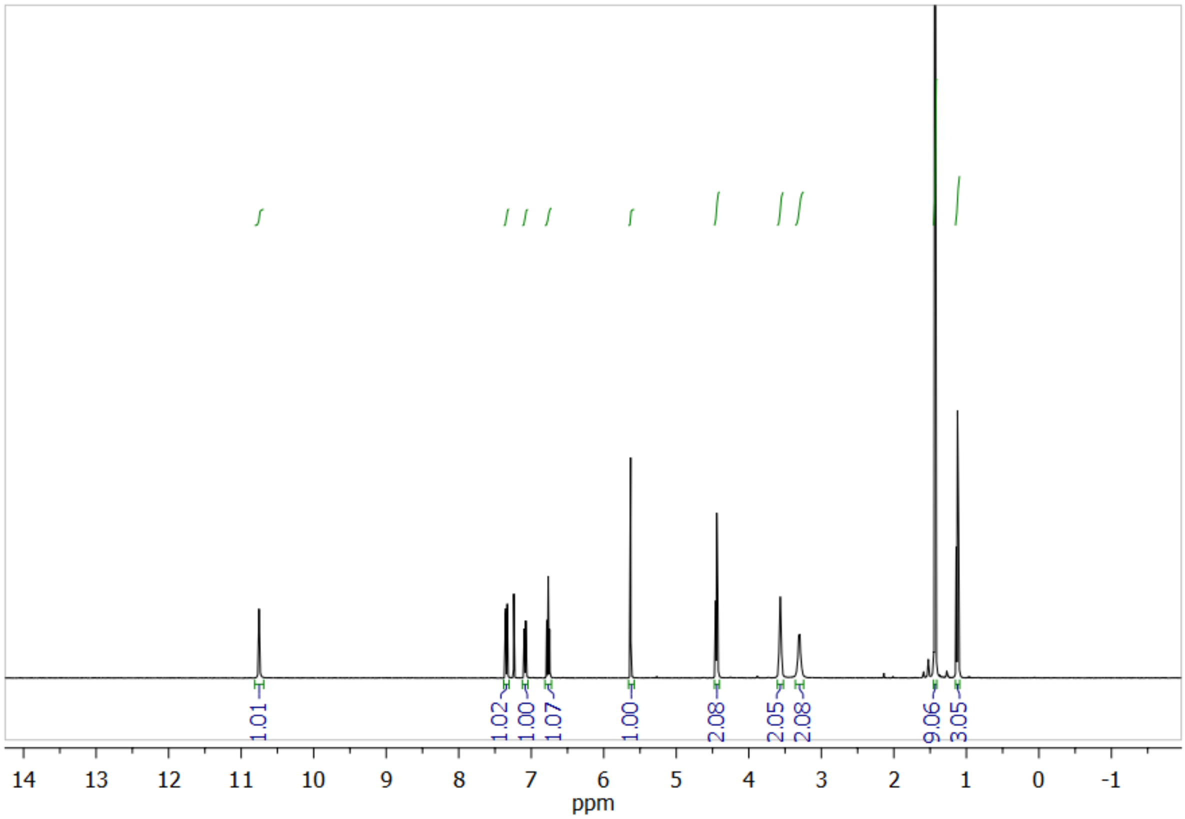


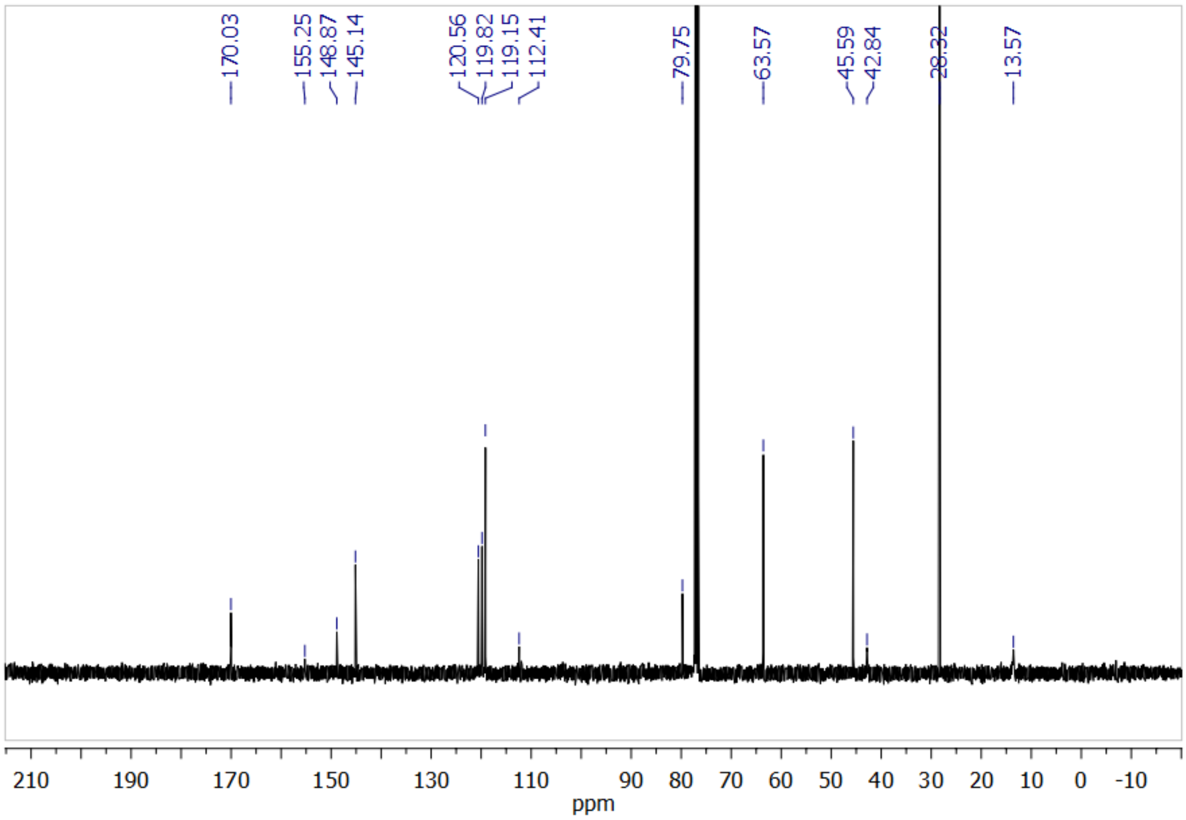

**2-((*tert*-Butoxycarbonyl)(isopropyl)amino)ethyl 2,3-dihydroxybenzoate (12c):**

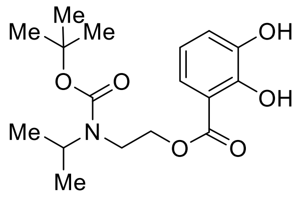


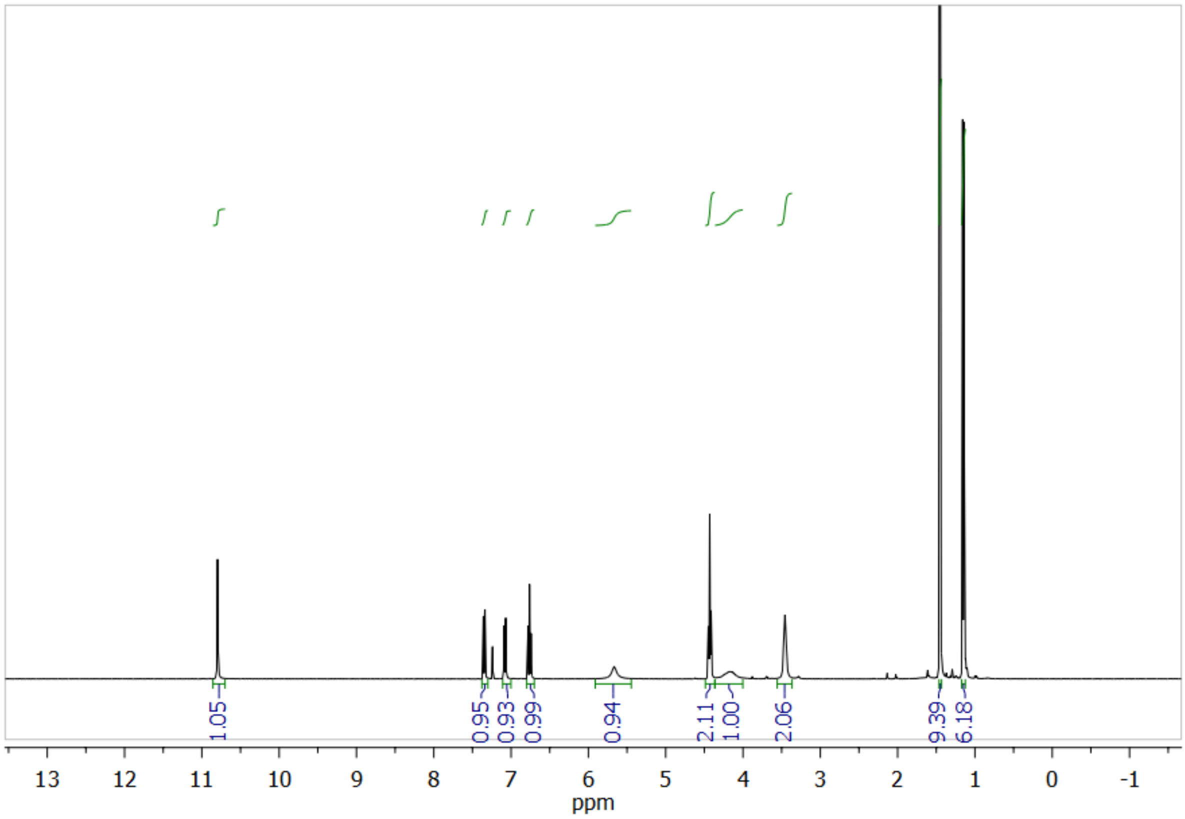


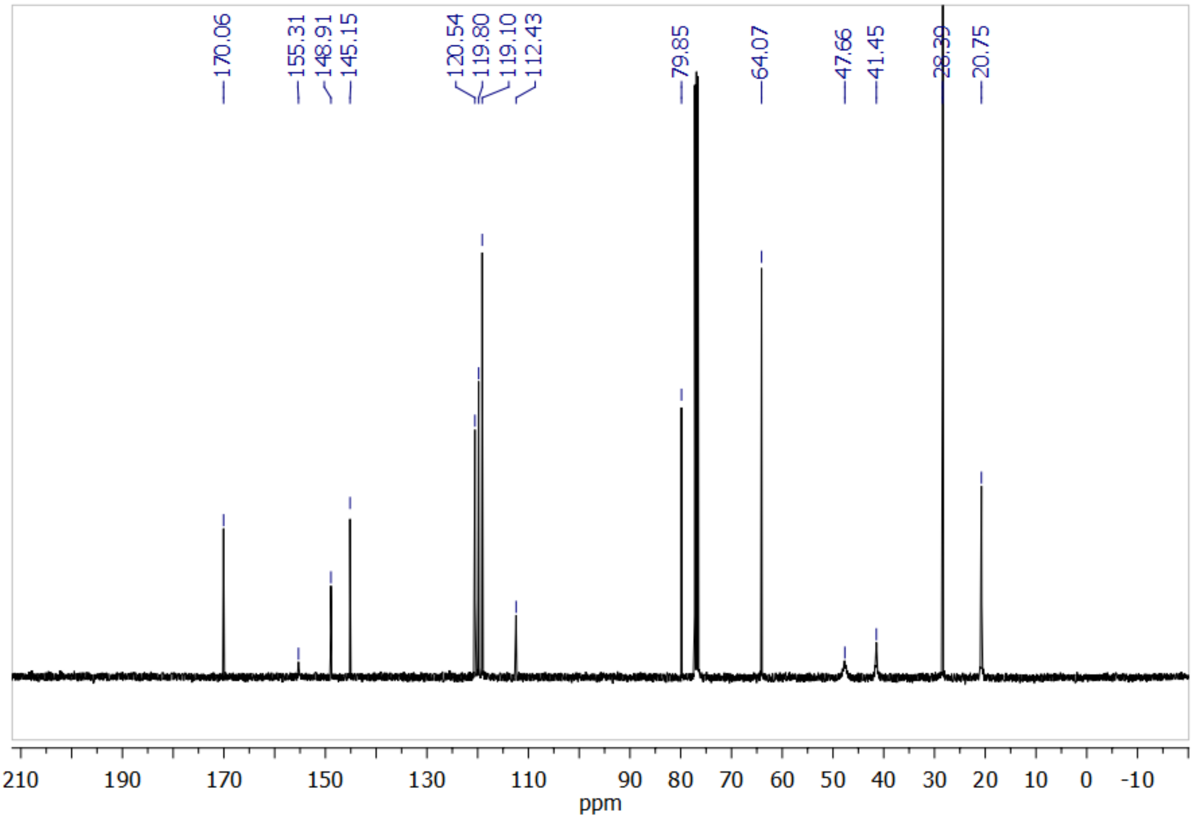

***tert*-Butyl 4-((2,3-dihydroxybenzoyl)oxy)piperidine-1-carboxylate (12d):**

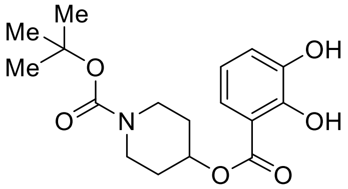


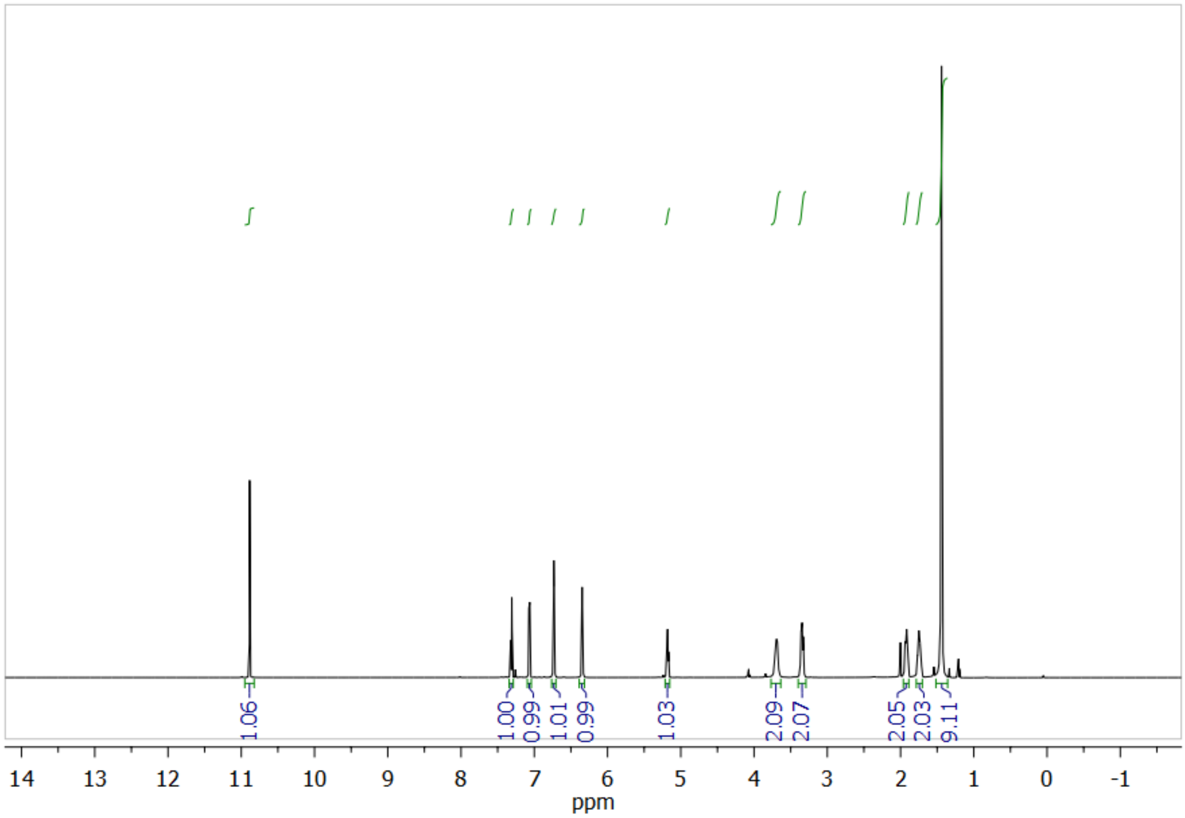


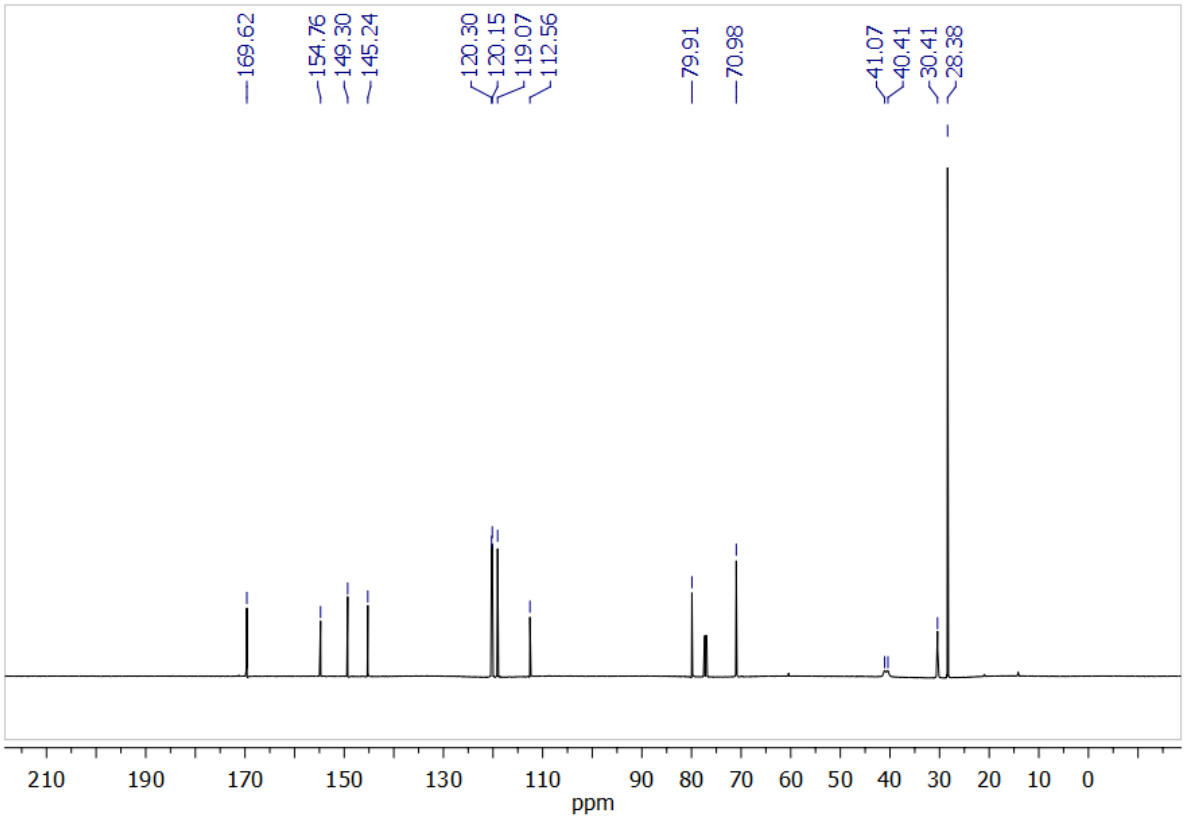

**2-(Methylamino)ethyl 2,3-dihydroxybenzoate hydrochloride (13a-$H_2$):**

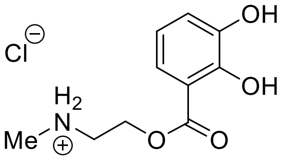


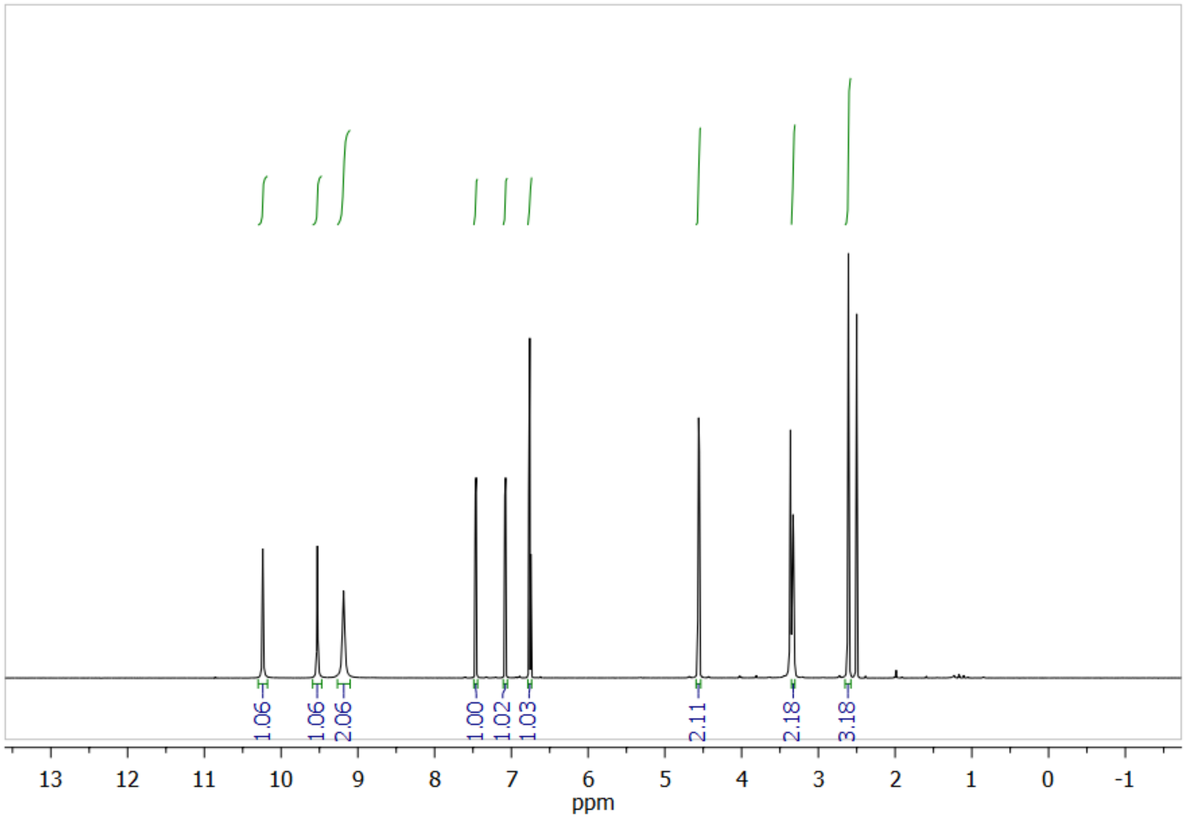


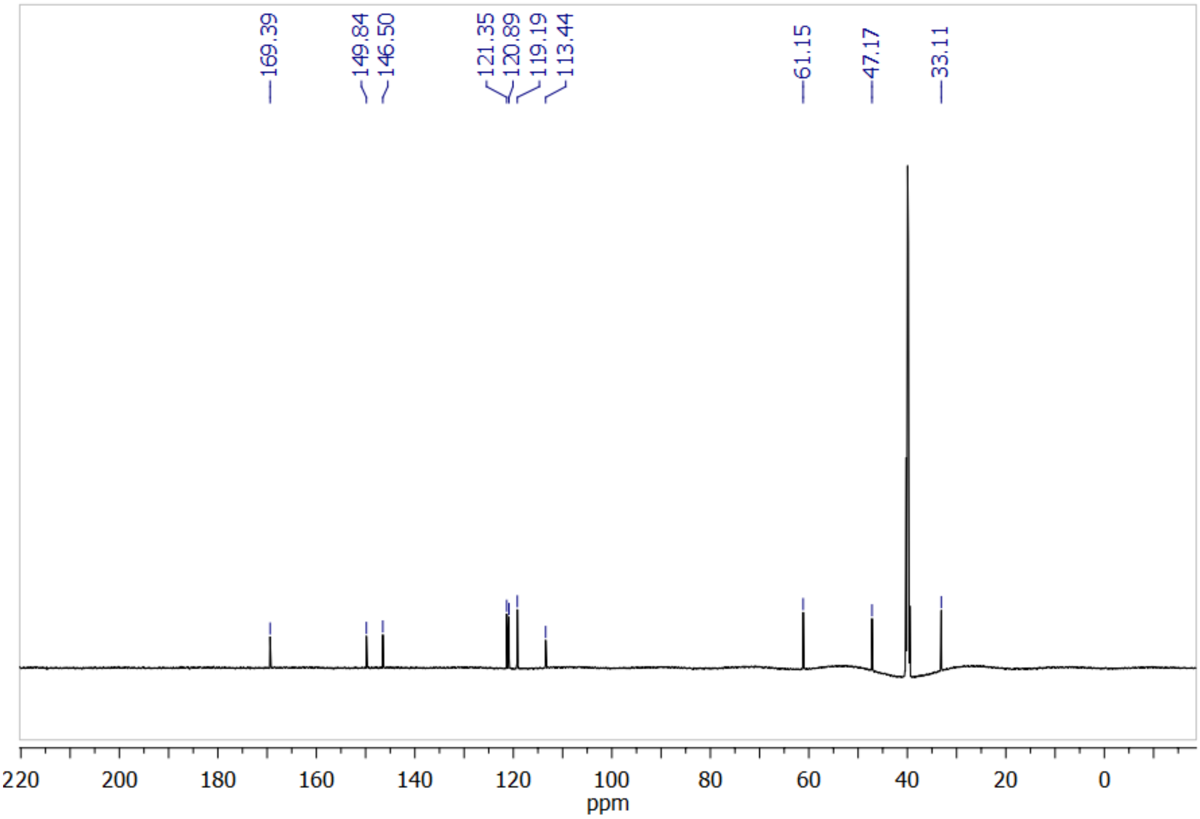

**2-(Ethylamino)ethyl 2,3-dihydroxybenzoate hydrochloride (13b-H₂):**

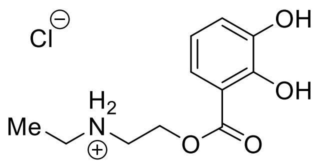


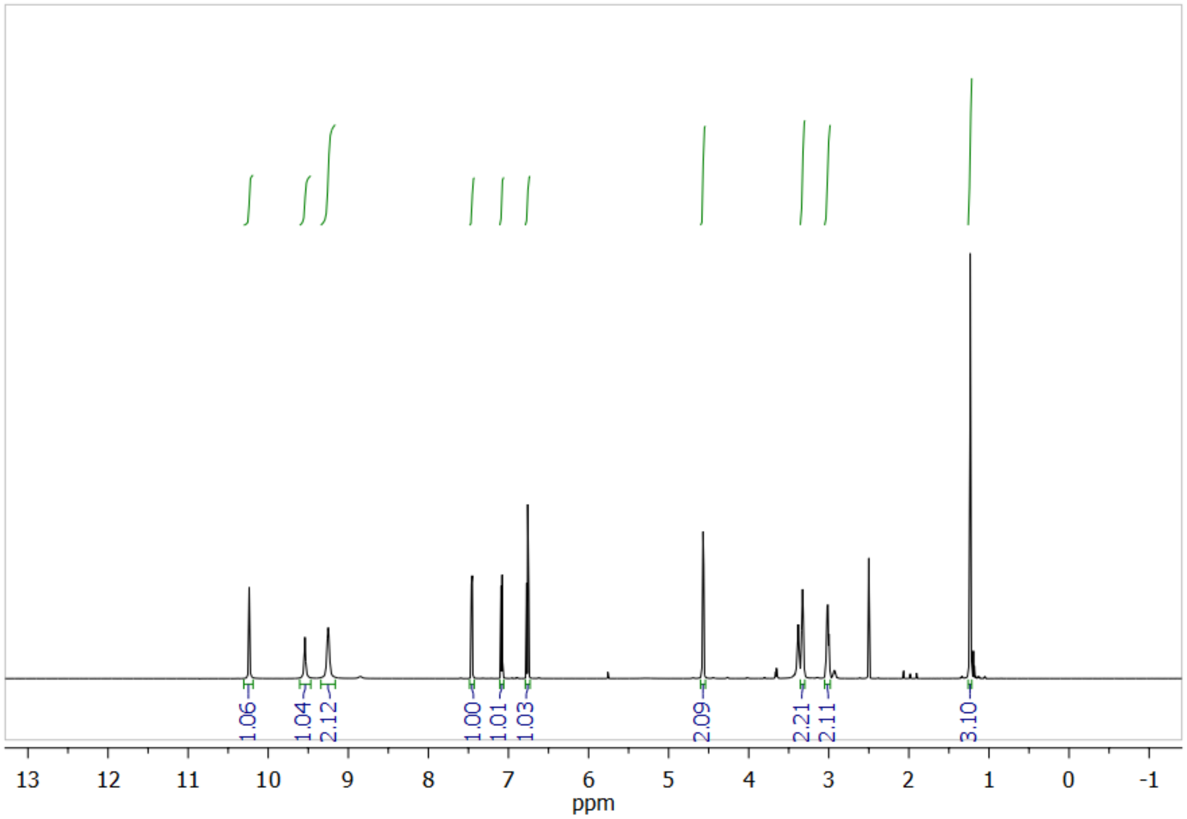


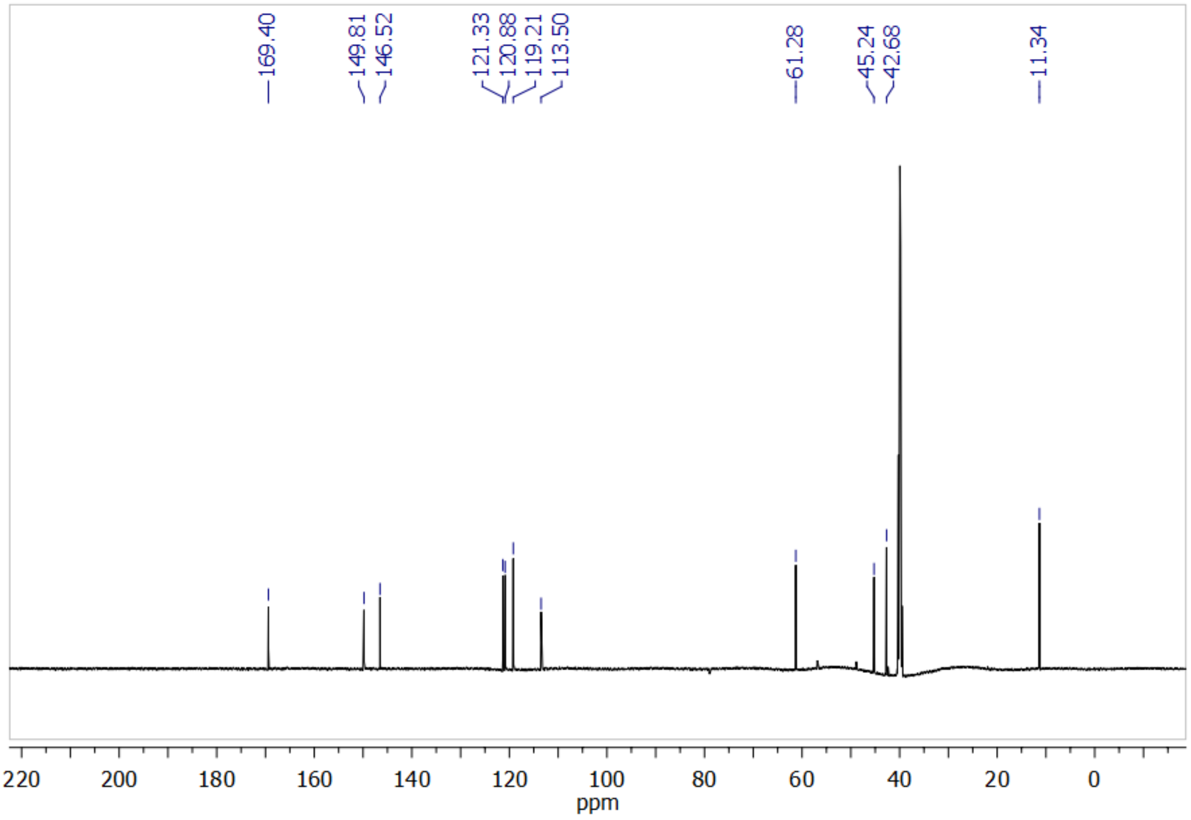

**2-(Isopropylamino)ethyl 2,3-dihydroxybenzoate hydrochloride (13c-H$_2$):**

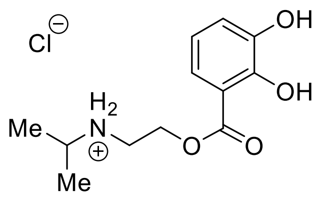


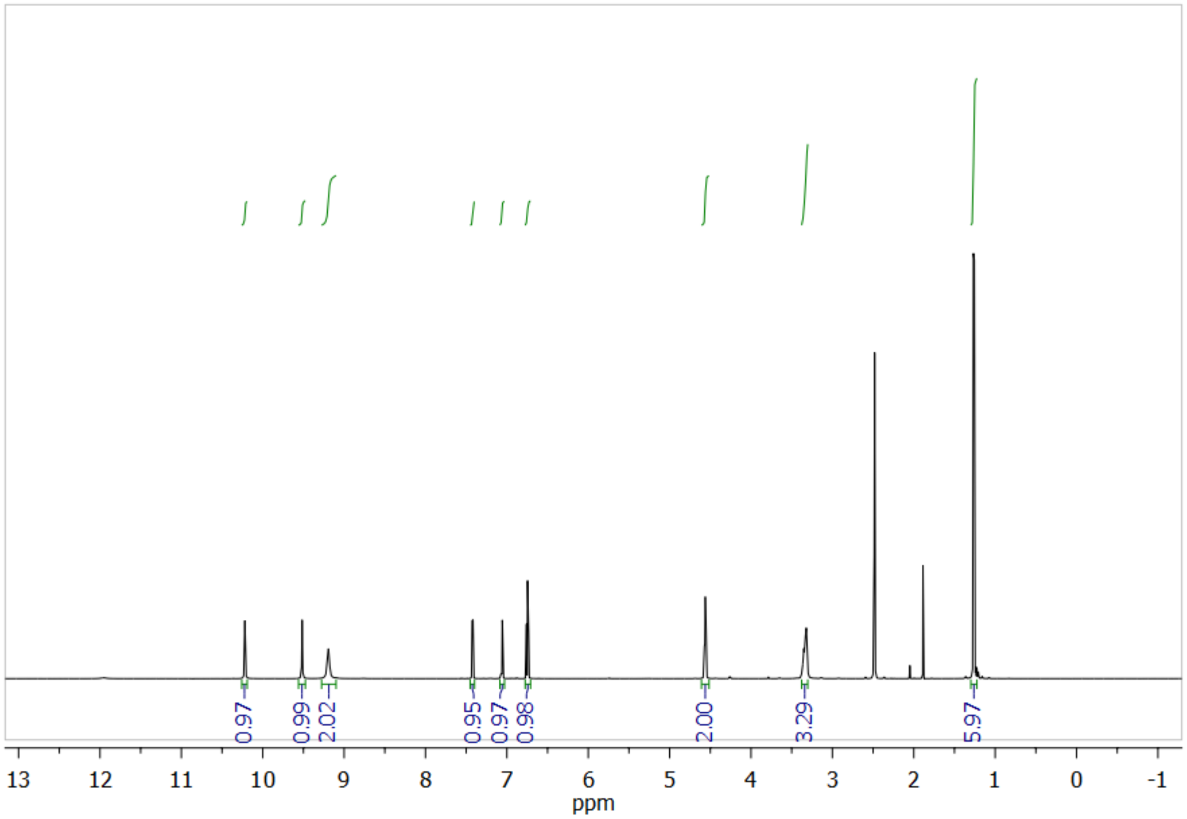


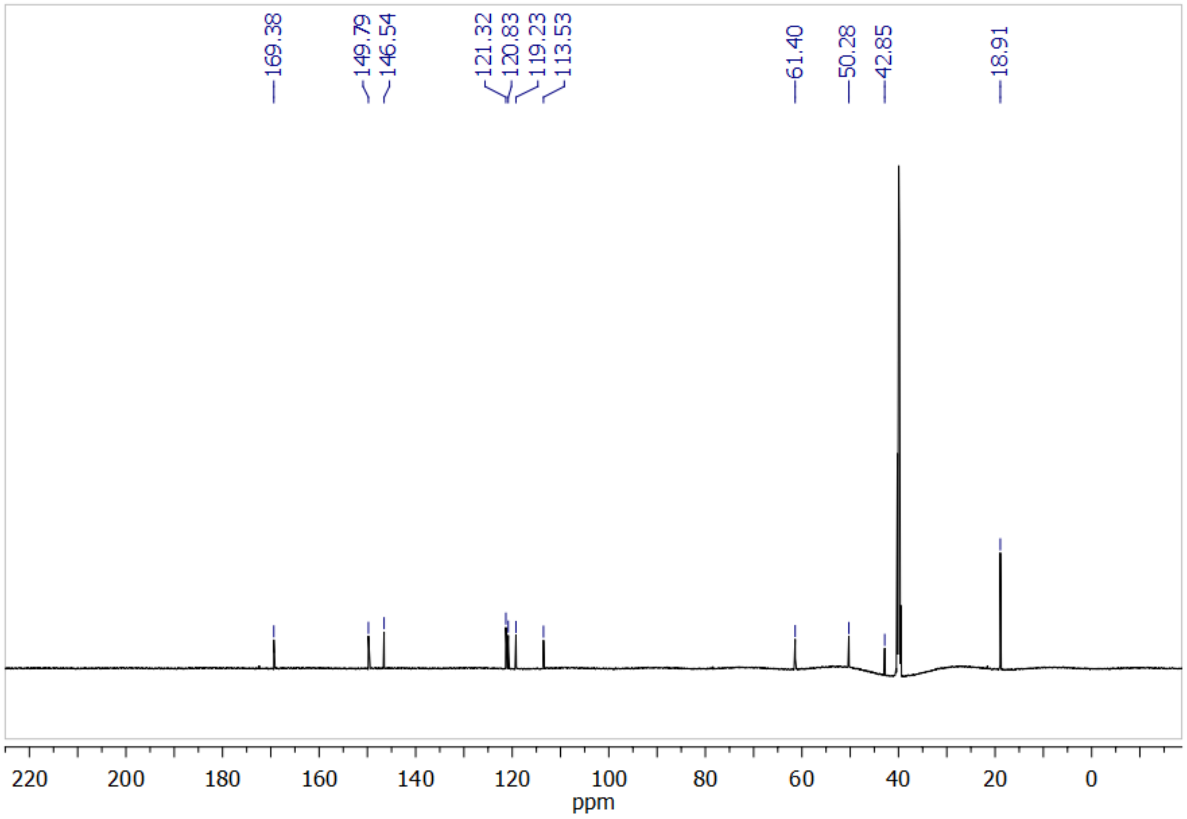

**Piperidin-4-yl 2,3-dihydroxybenzoate hydrochloride (13d-H2):**

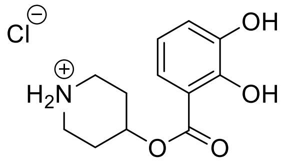


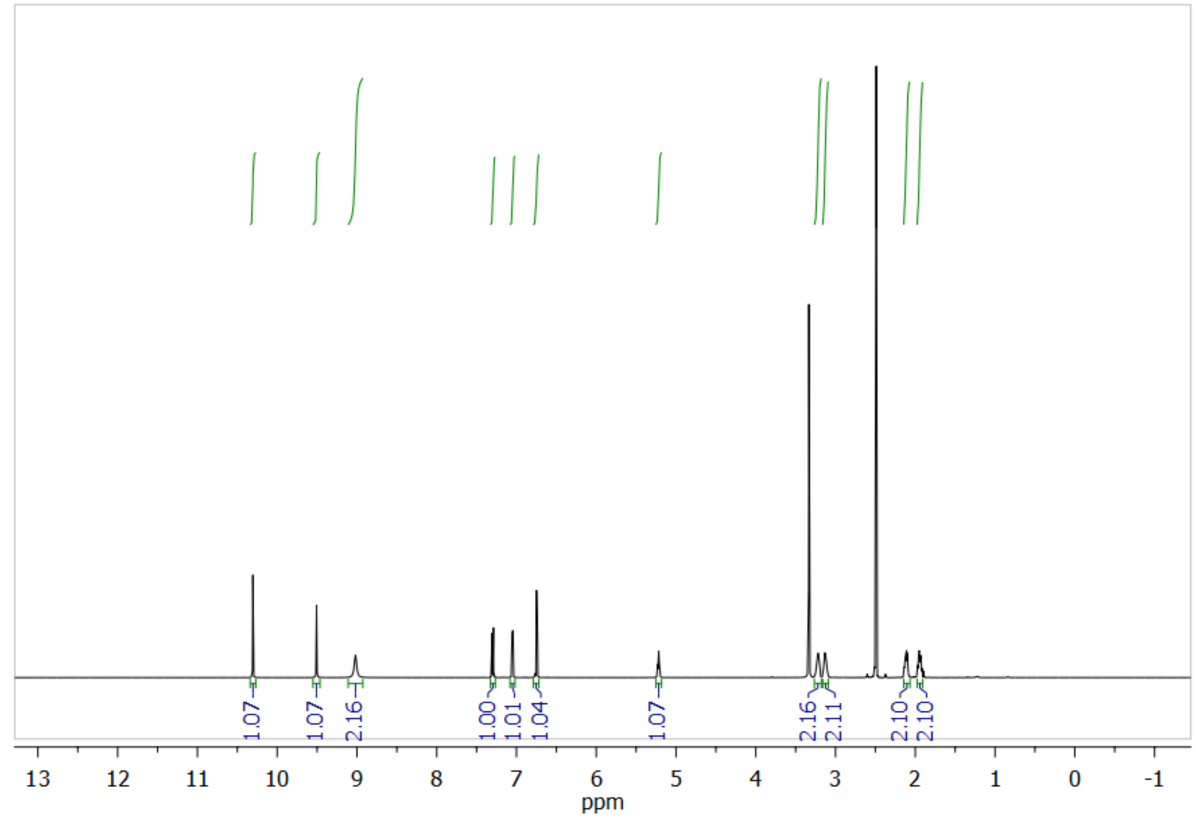


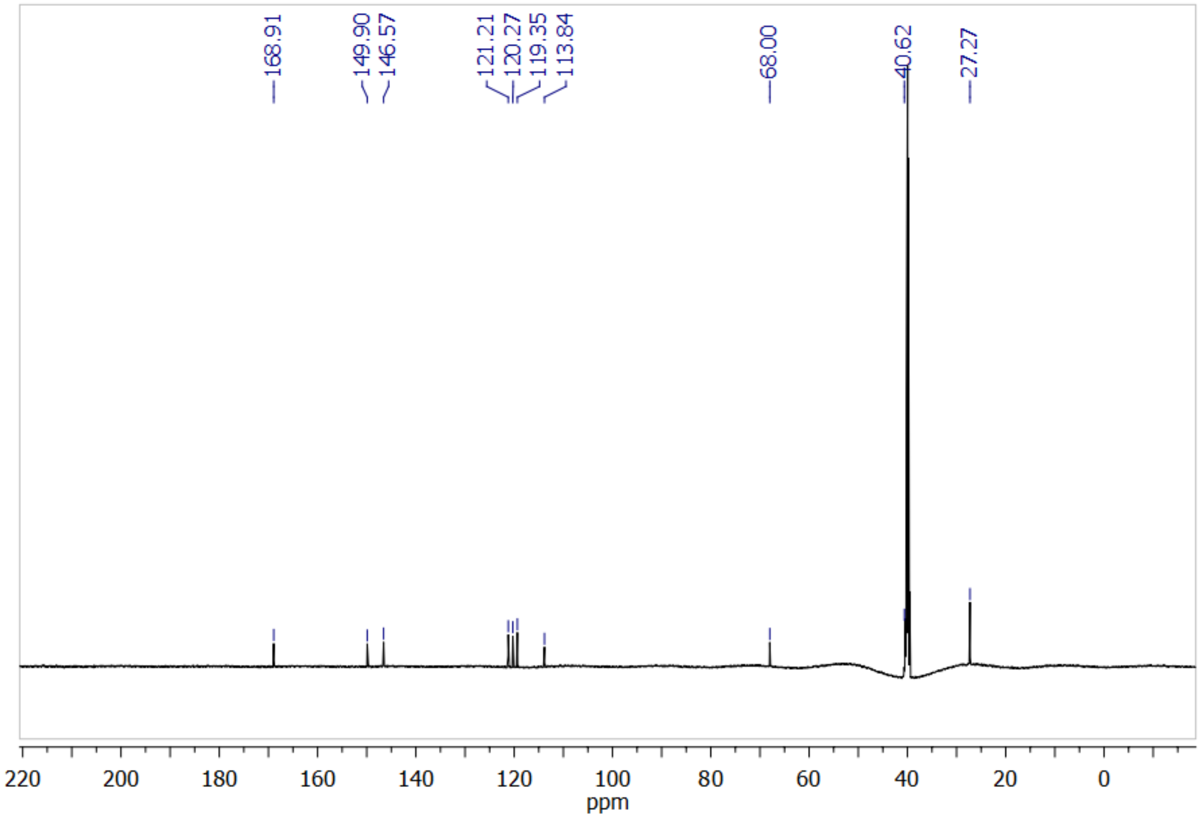

**References:**


1. Van Craen, D.; Albrecht, M.; Raabe, G.; Pan, F.; Rissanen, K. *Chem. Eur. J.* **2016**, *22*, 3255-3258.
2. Albrecht, M.; Isaak, E.; Baumert, M.; Gossen, V.; Raabe, G.; Fröhlich, R. *Angew. Chem.* **2011**, *123*, 2903-2906; *Angew. Chem. Int. Ed.* **2011**, *50*, 2850-2853.
3. Albrecht, M.; Isaak, E.; Moha, V.; Raabe, G.; Fröhlich, R. *Chem. Eur. J.* **2014**, *20*, 6650-6658.
4. Teitelbaum, A. M.; Meissner, A.; Harding, R. A.; Wong, C. A.; Aldrich, C. C.; Remmel, R. P. *Bioorg. Med. Chem.* **2013**, *21*, 5605-5617.
5. Albrecht, M.; Isaak, E.; Shigemitsu, H.; Moha, V.; Raabe, G.; Fröhlich, R. *Dalton Trans.* **2014**, *43*, 14636-14643.
6. (a) Krapcho, A. P.; Maresch, M. J.; Lunn, J. *Synth. Commun.* **1993**, *23*, 2443-2449. (b) Saari, W. S.; Schwering, J. E.; Lyle, P. A.; Smith, S. J.; Engelhardt, E. L. *J. Med. Chem.* **1990**, *33*, 97-101.
7. (a) Seebach, D.; Goliński, J. *Helv. Chim. Acta* **1981**, *64*, 1413-1423. (b) Blarer, S. J.; Schweizer, W. B.; Seebach, D. *Helv. Chim. Acta* **1982**, *65*, 1637-1654. (c) Hayashi, Y.; Gotoh, H.; Hayashi, T.; Shoji, M. *Angew. Chem. Int. Ed.* **2005**, *44*, 4212-4215. (d) Burés, J.; Armstrong, A.; Blackmond, D. G. *J. Am. Chem. Soc.* **2011**, *133*, 8822-8825.
8. Burés, J.; Armstrong, A.; Blackmond, D. G. *J. Am. Chem. Soc.* **2012**, *134*, 6741-6750.
9. Husmann, R.; Jörres, M.; Raabe, G.; Bolm, C. *Chem. Eur. J.* **2010**, *16*, 12549.
10. Van Craen, D. Hierarchisch gebildete Helicate als Plattform für stereoselektive Reaktionen und Katalyse. PhD Thesis. RWTH Aachen University; Aachen, Germany, 2017.
11. Chang, D.; Feiten, H.-J.; Engesser, K.-H.; van Beilen, J. B.; Witholt, B.; Li, Z. *Org. Lett.* **2002**, *4*, 1859-1862.
12. Isaak, E. Stereoselektive hierarchische Selbstorganisation von Helicaten. PhD Thesis. RWTH Aachen University; Aachen, Germany, 2013.
